# Defective transfer of parental histone decreases frequency of homologous recombination by increasing free histone pools in budding yeast

**DOI:** 10.1093/nar/gkae205

**Published:** 2024-03-30

**Authors:** Srinivasu Karri, Yi Yang, Jiaqi Zhou, Quinn Dickinson, Jing Jia, Yuxin Huang, Zhiquan Wang, Haiyun Gan, Chuanhe Yu

**Affiliations:** Hormel Institute, University of Minnesota, Austin, MN 55912, USA; Hormel Institute, University of Minnesota, Austin, MN 55912, USA; CAS Key Laboratory of Quantitative Engineering Biology, Guangdong Provincial Key Laboratory of Synthetic Genomics and Shenzhen Key Laboratory of Synthetic Genomics, Shenzhen Institute of Synthetic Biology, Shenzhen Institutes of Advanced Technology, Chinese Academy of Sciences, Shenzhen 518055, China; Hormel Institute, University of Minnesota, Austin, MN 55912, USA; Hormel Institute, University of Minnesota, Austin, MN 55912, USA; CAS Key Laboratory of Quantitative Engineering Biology, Guangdong Provincial Key Laboratory of Synthetic Genomics and Shenzhen Key Laboratory of Synthetic Genomics, Shenzhen Institute of Synthetic Biology, Shenzhen Institutes of Advanced Technology, Chinese Academy of Sciences, Shenzhen 518055, China; Division of Hematology, Department of Medicine, Mayo Clinic, Rochester, MN 55905, USA; CAS Key Laboratory of Quantitative Engineering Biology, Guangdong Provincial Key Laboratory of Synthetic Genomics and Shenzhen Key Laboratory of Synthetic Genomics, Shenzhen Institute of Synthetic Biology, Shenzhen Institutes of Advanced Technology, Chinese Academy of Sciences, Shenzhen 518055, China; Hormel Institute, University of Minnesota, Austin, MN 55912, USA

## Abstract

Recycling of parental histones is an important step in epigenetic inheritance. During DNA replication, DNA polymerase epsilon subunit DPB3/DPB4 and DNA replication helicase subunit MCM2 are involved in the transfer of parental histones to the leading and lagging strands, respectively. Single Dpb3 deletion (*dpb3Δ*) or Mcm2 mutation (*mcm2-3A*), which each disrupts one parental histone transfer pathway, leads to the other's predominance. However, the biological impact of the two histone transfer pathways on chromatin structure and DNA repair remains elusive. In this study, we used budding yeast *Saccharomyces cerevisiae* to determine the genetic and epigenetic outcomes from disruption of parental histone H3–H4 tetramer transfer. We found that a *dpb3Δ mcm2-3A* double mutant did not exhibit the asymmetric parental histone patterns caused by a single *dpb3Δ* or *mcm2-3A* mutation, suggesting that the processes by which parental histones are transferred to the leading and lagging strands are independent. Surprisingly, the frequency of homologous recombination was significantly lower in *dpb3Δ*, *mcm2-3A* and *dpb3Δ mcm2-3A* mutants, likely due to the elevated levels of free histones detected in the mutant cells. Together, these findings indicate that proper transfer of parental histones during DNA replication is essential for maintaining chromatin structure and that lower homologous recombination activity due to parental histone transfer defects is detrimental to cells.

## Graphical Abstract

**Graphical Abstract:**
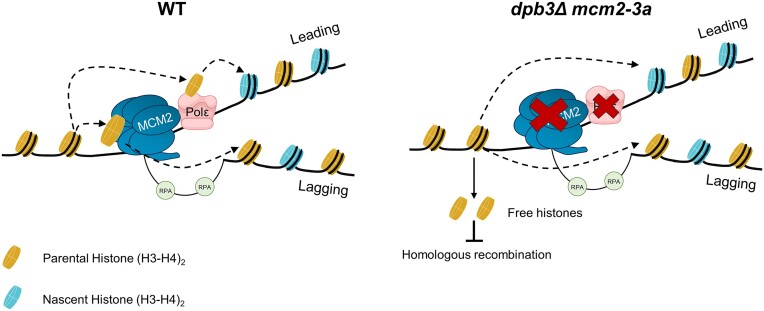

## Introduction

In eukaryotes, genomic DNA is packaged into an organized structure called chromatin. Each nucleosome core particle, which represents the basic unit of chromatin, is composed of two copies of each of the histones H2A, H2B, H3 and H4. These histones are assembled into an octameric core and tightly wrapped around a section of 147 bp of DNA (1). Upon the arrival of the DNA replication fork, the core histone octamer is disrupted. Following the DNA replication fork, the histone octamer is reassembled with both parental histones and newly synthesized histones (2,3). The recycling of each type of parental histone molecules behaves differently during DNA replication. The histone H3–H4 tetramers do not segregate and are transferred to new DNA (4). The recycling of histones H2A/H2B occurs independently of H3–H4 tetramer; and H2A–H2B dimers dissociate from the parental octamer and randomly reincorporate into newly assembled nucleosomes (5,6). The transfer of parental histone H3–H4 tetramers during chromatin replication is the essential first step in epigenetic inheritance (2,7). During every cell cycle, cells synthesize half the required histone molecules for DNA replication, with the other half coming from recycled parental histones (2,3). In yeast, the amount of parental H3–H4 tetramers transferred to the leading and lagging strands is nearly equal, though a slight bias exists for the lagging strand (8,9). DNA Polymerase ϵ, leading strand- specific (10,11), subunit Dpb3/Dpb4 mediates parental histone H3–H4 tetramer transfer to the leading strand (9). The Mcm2–Ctf4–Polα axis facilitates transfer of parental H3–H4 tetramers to the lagging strand. Specifically, MCM2, a subunit of the replicative helicase CMG, contains a histone-binding motif (HBM) that plays an essential role in mediating parental histone H3–H4 tetramer transfer (8,12,13). Mutations in the HBM, Ctf4, or Polα/primase that disrupt the CMG helicase's ability to connect to POLα, lead to a defect in transfer of the parental histone H3–H4 tetramer to the lagging strand (8,13). A recent study shows that the FACT (facilitates chromatin transcription) complex is also involved in parental histone recycle (14). Defects in the transfer of parental H3–H4 tetramers to either the leading or lagging strands compromise silencing at the *HML* locus in yeast and dysregulate gene expression in mouse embryonic stem cells (15,16). However, the impact of disrupting specific pathways for parental histone transfer on chromatin structure and DNA repair remains elusive.

Homologous recombination (HR) is an error-free mechanism for the DNA damage repair, which is essential to maintain genome stability. Previous studies have shown that HR is regulated by the chromatin environment (17). Several new histone H3–H4 chaperones are involved in this regulation. The chaperone CAF1 is conserved among all eukaryotes. It binds to the H3–H4 tetramer and mediates replication-coupled nucleosome assembly through interaction with PCNA (18,19). Caf1 mutants show increased homologous recombination in plants likely due to increased DNA accessibility (20). But in human cell lines, immunofluorescence staining showed that CAF1 is locally recruited to DNA double strand break sites (21). The chaperone ASF1 presents the newly synthesized histone H3–H4 to Rtt109–Vps75 for acetylation of histone H3K56, after which the ASF1-bound H3–H4 is transferred to CAF1 or RTT106 (22,23). CAF1 and ASF1 promote homologous recombination through nucleosome assembly (24,25). These studies suggest a connection between the new histone chaperones and HR. More recently, the parental histone deposition pathways have been implicated in template switch recombination, an error-prone of HR, in yeast (26). It is still unclear whether there is a connection between parental histone chaperones and HR.

In this study, we compared a *dpb3Δ mcm2-3A* double mutant to single Dpb3 and Mcm2 mutants, as well as the wild-type (WT) strain, in experiments characterizing the strand bias of parental histone transfer, chromatin structure, genomic instability, and HR. We show that the leading and lagging strand parental histone transfer process are independent as the double mutant (*dpb3Δ mcm2-3A*) neutralizes the single mutant's asymmetric parental histone pattern. The homologous recombination frequency is significantly decreased in *mcm2-3A*, *dpb3Δ* and the double mutants, likely due to elevated free histone level. Our findings establish a connection between the parental histone transfer process and the regulation of DNA repair pathways.

## Material and methods

### Yeast strains

All *S. cerevisiae* yeast strains were of the W303-1A genetic background (MATa leu2-3 112 trp1-1 ura3-1 his3-11 ade2-1 can1-100) and are listed in Supplementary Table S1. To create the strains, deletions, tagging, and mutagenesis were performed using polymerase chain reaction (PCR)–based methods or CRISPR-Cas9, as previously reported (13,27,28). The single *mcm2-3A* mutant has three-point mutations in the histone H3 binding domain, which disrupt the ability of parental histone H3 to bind to replicating DNA strands (12).

### eSPAN

We have previously used eSPAN to detect binding patterns of a targeted protein on leading or lagging DNA strands (29–31). In this study, we used H3K4me3, one of the most abundant histone methylation marks, to track parental histones during S-phase, because histone methylation of newly formed nucleosomes gradually accumulates during late S/G2-phase (9,13,32). We used H3K56ac to track newly synthesized histone H3 in S-phase, because in S-phase it is present only on newly synthesized histones; it is completely removed during G2-phase (33,34).

We performed eSPAN following a previously described procedure (30,32). WT (cyc560), *mcm2-3A* (cyc552), *dpb3Δ* (cyc604), *dpb3Δ mcm2-3A* (cyc602) and other related strains (Supplementary Table S1) were used. Specifically, yeast cells were grown in YPD medium (1% yeast extract, 2% peptone, 2% glucose) to exponential growth phase. Cells were then arrested in G1-phase using two doses of α factor (5 μg/ml; EZBiolab) for three hours at 25°C. Sample was released into fresh YPD medium containing 400 mg/l BrdU and 200 mM hydroxyurea for 45 min at 30°C. Hydroxyurea stalls the replication fork but does not interfere with the process of transferring newly synthesized and parental histones (9,13).

Cells were fixed by adding freshly prepared paraformaldehyde (to 1%) at 25°C for 20 min, followed by quenching with 0.125 M glycine for 5 min at room temperature. After fixation, cells were washed twice with cold water and harvested by centrifugation at 3000 rpm for 5 min. After harvesting, cells were washed and lysed in 0.1 ml ChIP lysis buffer (50 mM HEPES [pH 8.0], 150 mM NaCl, 2 mM EDTA, 1% Triton X-100, 0.1% sodium deoxycholate) with glass beads. Lysate was harvested and washed twice with NP buffer (1.6 M sorbitol, 2 mM CaCl_2_, 5mM MgCl, 50 mM NaCl, 14 mM β-mercaptoethanol, 10 mM Tris–HCl [pH 7.4], 0.075% NP-40, 5 mM spermidine). The chromatin pellet was digested with Micrococcal Nuclease (MNase) (LS004797, Worthington) in 350 μl NP buffer at 37°C for 20 min into primarily di- and mononucleosomes. The digestion was terminated with 5 μl 0.5 M EDTA and 90 μl 5× ChIP lysis buffer and was kept on ice for 30 min. Cells were then lightly sonicated for three cycles (Bioraptor Pico machine, 30 s ON/OFF) at 4°C to release chromatin fragments into solution. Soluble chromatin (eSPAN samples) was immunoprecipitated with anti-H3K4me3 antibody (ab8580 Abcam) or anti-H3K56ac antibody (34). Protein G Sepharose beads (17-0618-02, GE Healthcare) was used to recover the targeted chromatin. After washing the beads extensively, DNA from ChIP was recovered using the Chelex-100 protocol (35).

ChIP DNA was denatured by incubating it at 100°C for 5 min and then immediately cooling it on ice for 5 min. DNA was diluted with BrdU IP buffer (1× PBS, 0.0625% Triton X-100 [v/v]). BrdU antibody (0.17 μg/ml; 555627, BD Biosciences) was added and samples were incubated at 4°C for 2 h. Next, 20 μl Protein G beads (17-0618-02, GE Healthcare) were added to each sample and incubated for an additional hour at 4°C. The beads were extensively washed, DNA was eluted with 100 ul 1× TE buffer (pH 8.0, 10mM Tris–HCl, 1 mM EDTA) containing 1% SDS and purified using a QIAGEN MinElute PCR Purification kit. ssDNA libraries were prepared using an Accel-NGS 1S Plus DNA library kit (10096, Swift Biosciences). For ChIP and eSPAN experiments, each experiment is repeated at least once.

### Sequence mapping and data analysis

The sequence mapping, nucleosome mapping, and eSPAN analysis was performed as previously described (9,13). Briefly, the reads were mapped back to the Saccharomyces Genome Database (http://www.yeastgenome.org/) reference genome with the Bowtie2 software (36). Only paired-end reads with both ends mapped correctly were selected for continued analysis. 120–170 bp DNA fragments calculated by the paired-reads were used to obtain the nucleosome occupancy by self-developed Perl programs. To calculate the eSPAN bias pattern, the forward (Watson strand) and reverse (Crick strand) reads following the reference genome were separated by self-developed Perl programs. The nucleosomes position around DNA replication origins were previously determined (37). Total eSPAN sequence reads at ± 10 nucleosomes surrounding the DNA replication origins were counted for separate strands. The log_2_ ratio of Watson strand reads over Crick strand reads at each nucleosome position was used to obtain the average bias pattern of eSPAN. All program scripts will be thoroughly made available to any non-commercial requester upon request.

### Analysis of silencing-loss at the *HML* locus using the CRASH assay

WT (JRY10790), *mcm2-3A* (cyc853), *dpb3Δ* (cyc756), *dpb3Δ mcm2-3A* (cyc777) and *cac1Δ* (cyc754) strains were used to measure the apparent silencing-loss rate at the *HML* locus. Briefly, 10 colonies of each strain were grown separately in YPD medium to saturation, diluted to OD600 = 0.01 in YPD, and grown for 5 h at 30°C. For the GFP positive control, cells were treated with 20 μM nicotinamide; for the RFP positive control, cells were grown in hygromycin (200 μg/ml). The apparent silencing-loss rate at the *HML* locus was calculated by dividing the number of RFP+ GFP+ cells (cells that have recently undergone Cre-mediated recombination express GFP but not RFP) by the total number of cells with the potential to lose silencing (RFP+ GFP– and RFP+ GFP+). For each colony, 50 000 events were analyzed using a BD Fortessa cytometer.

### Chromatin fractionation

We performed chromatin fractionation following a previously published procedure (Figure 5A) (38). WT (cyc614), *mcm2-3A* (cyc933), *dpb3Δ* (cyc931), *dpb3Δ mcm2-3A* (cyc929) *rad53Δ* (cyc939) and other related strains (Supplementary Table S1) were used. Cells were grown to OD600 = 0.5–0.6, arrested at G1-phase using two doses of α factor (5 μg/ml), grown for 3 h at 25°C, and released in fresh YPD medium for 50 min, yielding late S-phase cells. The cells were washed with cold water and harvested by centrifuging samples at 3000 rpm for 5 min. Harvested cells were washed with spheroplast buffer (0.6M sorbitol, 24 mM Tris [pH 7.5]), suspended in the same buffer supplemented with Zymolase (0.5 mg/ml, L2524, Sigma), and incubated at 30°C with gentle shaking. Spheroplasts were then washed with cold spheroplast buffer and suspended in 425 μl spheroplast buffer and 50 μl 10× lysis buffer (500 mM potassium acetate, 20 mM MgCl_2_, 200 mM HEPES [pH 7.5]), along with protease inhibitors (Roche). Spheroplasts were lysed by adding 20 μl 20% triton X-100 and kept on ice for 10 min. Soluble and chromatin-bound histones were separated via centrifugation at 12 000 g for 15 min at 4°C. The soluble and chromatin fractions were probed with anti-HA (12CA5, Sigma for H3 detection), anti-H3K4me3 (ab8580, Abcam) and anti-PGK1 (459250, Thermofisher) antibodies. For the chromatin fractionation experiments, each experiment is repeated at least twice.

### Chromatin accessibility assay using MNase

MNase preferentially digests the naked DNA between nucleosomes and is commonly used to probe chromatin structure. To measure chromatin accessibility using MNase, 100 ml log phase cells were washed with cold water and harvested by centrifugation. Spheroplasts were prepared by suspending cells in spheroplast buffer (1 M sorbitol, 0.5 mg/ml Zymolase, 2 mM β-mercaptoethanol) at 30°C for 20 min. Spheroplasts were washed twice with sorbitol wash buffer (1 M sorbitol, 1 mM PMSF, 2 mM β-mercaptoethanol), suspended in 600 μl spheroplast digestion buffer (1 M sorbitol, 50 mM NaCl, 10 mM Tris–HCl [pH 7.5], 5mM MgCl_2_, 1 mM CaCl_2_, 1mM β-mercaptoethanol, 0.075% v/v NP-40), and divided into 200 μl aliquots containing varying concentration of MNase (Worthington Biochemical) for 10 min at 37°C. Reactions were terminated by adding 20 μl stop solution (5% SDS, 250 mM EDTA) followed by proteinase K digestion for 2 h at 55°C. DNA was extracted twice with phenol: chloroform, and RNA was degraded by treating with RNAase A for 1 h at 37°C. DNA was suspended in TE buffer (10 mM Tris pH 8.0, 1mM EDTA), and digested nucleosomes were separated using 2% agarose gel. For MNase digestion experiments, each experiment is repeated at least twice.

### Rad52-YFP foci counting

In budding yeast, Rad52 forms spontaneous foci, predominantly during the S- and G2-phases of the cell cycle. These foci are believed to be sites of DNA lesion repair (39). WT (cyc949), *mcm2-3A* (cyc943), *dpb3Δ* (cyc945), and *dpb3Δ mcm2-3A* (cyc947) and other related strains are listed in Supplementary Table S1. We counted Rad52-YFP foci in yeast strains as previously described (34,39). Briefly, 1.5 ml cultures of yeast strains expressing Rad52-YFP grown at 25°C were harvested, washed twice with SCM-TRP, and resuspended in SCM-TRP. Living cells were then immobilized on glass slides and images were acquired using an LSM10 confocal microscope equipped with a Plan-Apochromat 40×/0.95 oil lens. All focal planes were analyzed, and cells with Rad52-YFP foci from one imaging field were counted as positive.

### Western blot to detect yeast γ-H2AX and Rad53-P

To detect γ-H2AX, 10 ml log phase cultures of yeast grown in YPD medium with or without 0.1% methylmethane sulphonate (MMS) were harvested and washed with 20% TCA. Cell pellets were resuspended in 250 μl 20% TCA, followed by lysis with 0.5 ml glass beads. The glass beads were removed, 0.3 ml 5% TCA was added, and precipitated proteins were collected by centrifugation. Samples were transferred to 1.5 ml tubes and 0.7 ml 5% TCA was added. Cells were centrifuged at 13 000 rpm at 4°C for 10 min and supernatant was discarded. The cell pellet was washed with 100% cold ethanal and resuspended in 100 μl SDS loading buffer with 50 μl 1 M Tris pH 8.0. After 10 min at 95°C, insoluble material was removed by centrifugation and the supernatant was fractionated by SDS-PAGE. The sample collection and process with Rad53-P followed previous publication (40). Blotting was then performed with anti-γ-H2A antibody (ab15083, Abcam) or Rad53 antibody (ab104232, Abcam). For western blot experiments, each experiment is repeated at least once.

### Measuring HR frequency

To measure HR frequency, we first transformed yeast strains with a DNA fragment containing a neomycin (G418) resistance gene (*Neo*). The *Neo* flanking sequence was 100% homologous to the yeast *URA3* locus (Figure 6A). Successful HR generated colonies resistant to both G418 and FOA (5-fluoroorotic acid). WT (cyc876), *mcm2-3A* (cyc892), *dpb3Δ* (cyc881), *dpb3Δ mcm2-3A* (SK66) and other related strains (Supplementary Table S1) were used. Rad52 is required for HR. Thus, we also included a *rad52Δ* mutant as a negative control.

In addition, we quantified HR using an *in vivo* LU system (Figure 6C) (41). In this system, a 2.5-kb repeat sequence is inserted into the *Leu2* gene to disrupt its function. However, HR between the repeat sequences can restore the function of Leu2. WT (YLD87), *mcm2-3A* (SK37), *dpb3Δ* (SK36), *dpb3Δ mcm2-3A* (SK38) and other related strains (Supplementary Table S1) were used. In the experiment, six independent colonies for each strain studied were picked from fresh-cultured YPD plate (or SC-Ura plate), resuspended in water, and plated on SC-Leu (or SC-Ura-Leu), or YPD (or SC-Ura) to determine the number of Leu+, or viable colonies, respectively. The median frequency of recombination for each strain was calculated per viable cell number (determined on YPD or SC-Ura).

## Results

### A symmetric distribution of parental histones H3–H4 at replicating DNA strands is detected in *dpb3Δ mcm2-3A* double mutant cells

**Figure 1.**
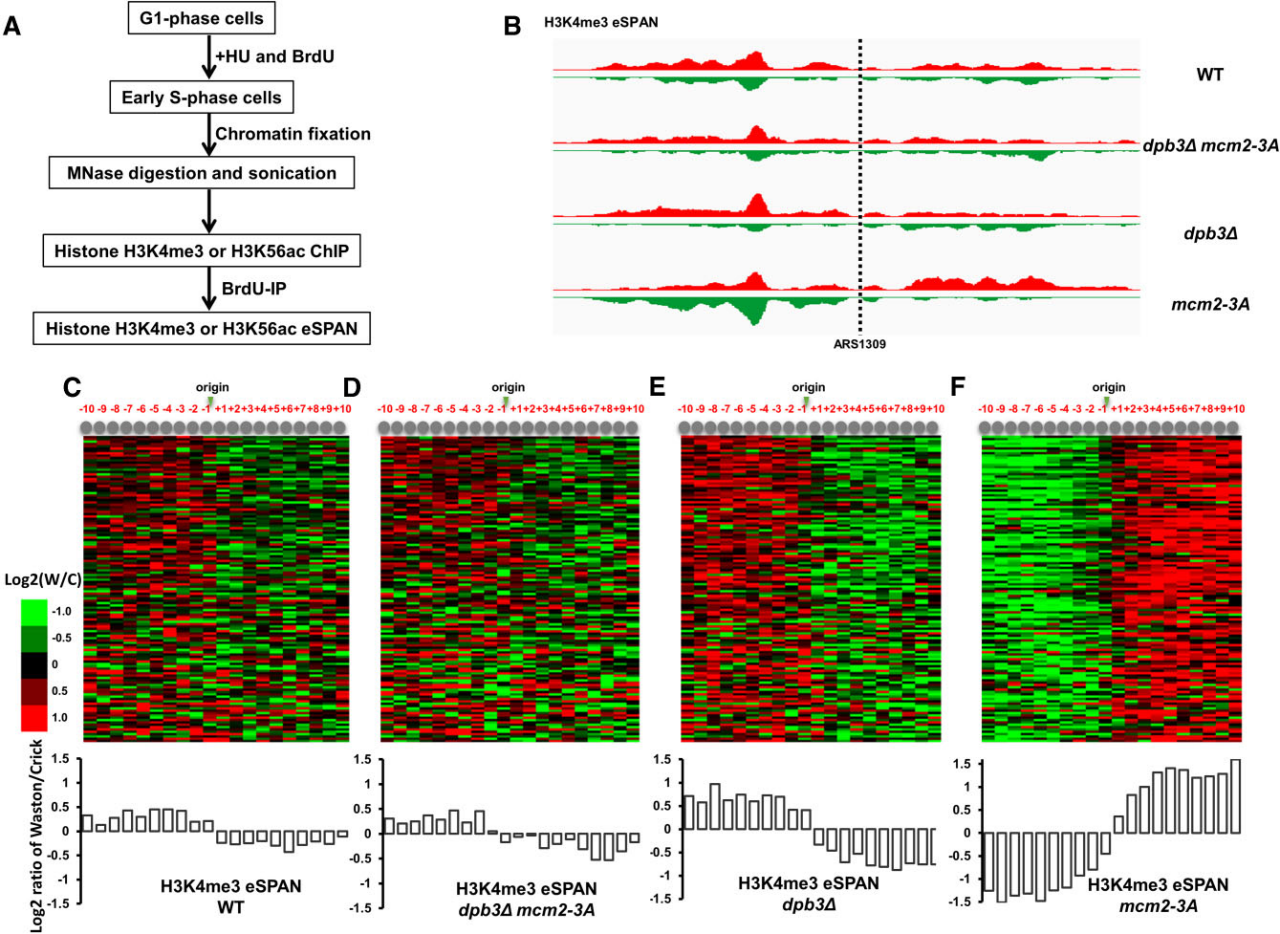
Combination of *dpb3Δ* and *mcm2-3A* mutations neutralizes single *dpb3Δ* or *mcm2-3A* mutants’ strand bias in transferring parental histone H3–H4 tetramers during DNA replication. (**A**) Procedure for monitoring the deposition of parental (H3K4me3) and newly synthesized (H3K56ac) histone H3 at early replication origins. (**B**) Snapshot of parental histone H3 (H3K4me3) eSPAN reads enrichment at leading and lagging strands at the early replication origin ARS1309 for wild-type (WT), *dpb3Δ*, *mcm2-3A* and *dpb3Δ mcm2-3A* strains. The sequence reads were mapped to both the Watson strand (red) and the Crick strand (green) of the reference genome. (**C–F**) *Top*: heatmaps representing the bias ratio of parental histone H3 (H3K4me3) eSPAN peaks for WT, *dpb3Δ*, *mcm2-3A* and *dpb3Δ mcm2-3A* strains at each of the 10 individual nucleosomes surrounding each of the 134 early DNA replication origins. Individual nucleosomes are represented by the circles at the top of the heatmaps, and their positions are indicated relative to the origin (−10 to + 10). Each row represents the average log_2_ Watson/Crick ratio of H3K4me3 eSPAN sequence reads at one origin. *Bottom*: average bias ratio of parental histone H3 (H3K4me3) eSPAN peaks for WT, *dpb3Δ*, *mcm2-3A* and *dpb3Δ mcm2-3A* strains at each of the 10 nucleosomes surrounding the 134 early replication origins.

To detect whether interference can take place between the two nucleosome assembly pathways, we generated a double mutant strain *dpb3Δ mcm2-3A*. We then used enrichment and sequencing of protein-associated nascent DNA (eSPAN) to analyze the binding of parental and newly synthesized H3–H4 tetramers to leading and lagging DNA strands in the *dpb3Δ mcm2-3A* strain (Supplementary Figure S1). Hydroxyurea was used to arrest synchronized cells in early S-phase (Figure 1A) (29), after which H3K4me3 was used to track parental histone H3, and H3K56ac was used to track newly synthesized histone H3 (Supplementary Figure S1). eSPAN mapping snapshots are displayed at Figure 1B and Supplementary Figure S2A.

As previously reported (9), parental histone H3–H4 tetramers in WT cells showed a slight lagging strand bias for all early S-phase replication origins (Figure 1C–F). The parental tetramer in the *dpb3Δ* mutant displayed lagging strand bias, whereas the *mcm2-3A* mutant displayed leading strand bias, which is consistent with previous reports (9,13). In the *dpb3Δ mcm2-3A* double mutant, the parental tetramer exhibited a slight lagging strand bias, similar to that of the WT cells (Figure 1C, D). Newly synthesized histone H3–H4 tetramers showed the reverse pattern of parental H3–H4 (Supplementary Figure S2). These results suggest that no interaction exists between the two parental histone transfer pathways. If one pathway is defective, the other becomes predominant. In the next, we tested whether the previous identified new H3–H4 tetramer chaperones (Caf1, Asf1 and Rtt106) are also involved in parental histone transfer. Caf1 is a chaperone for newly synthesized histones, and Cac1 is its large subunit (7). In this effort, we used the *cac1Δ*, *asf1Δ* and *rtt106Δ* mutants. The H3K4me3 eSPAN results indicated that parental histones are still transferred to both leading and lagging strand with a little bias to the lagging strand (Supplementary Figure S3). This pattern is very similar to the wild type cells (Figure 1C). This result also indicated that the new histone chaperones (Caf1, Asf1 and Rtt106) are likely not involved in parental histone deposition. This result aligns with a recent report indicating that Caf1 is not involved in parental histone transfer in the mammalian cell line system (42).

Here, we used the CRASH (Cre-reported altered states of heterochromatin) assay to monitor the transient loss of heterochromatin silencing at the *HML* locus during cell division (i.e. strains’ epigenetic instability phenotype) (43,44). We observed that the *dpb3Δ* and *mcm2-3A* double mutant shows an additive effect (Supplementary Figure S4A, B). These results are similar to those reported previously (16). Next, we compared epigenetic instability in these mutants to that in a Caf1 mutant (*Δcac1*). As previously reported, epigenetic instability in the *cac1Δ* mutant was much greater than in the parental histone chaperone mutants (Supplementary Figure S4A, B) (44). Similar to *cac1Δ*, the *asf1Δ* and *rtt106Δ* also shows a much higher percentage of loss of silencing than the parental histone H3–H4 chaperone mutants in the previously published data (16,44). Taken together, our data and previously published finding suggest that new histone assembly pathway has a greater impact on epigenetic integrity than the parental histones transfer pathway at *HML* locus. In conclusion, our eSPAN and phenotype data indicate that the degree of parental histone strand bias is not directly correlated with epigenetic instability at *HML* locus (see more in Discussion part).

### The impact of parental histone transfer pathways on chromatin structure

As parental histone recycling is a fundamental process in all eukaryotic cells, we investigated whether defects in these pathways had phenotypic consequences other than the loss of silencing states. First, we examined DNA replication kinetics using fluorescence activated cell sorting (FACS). DNA replication kinetics in the *mcm2-3A* strain were equivalent to those in WT cells (Supplementary Figure S5), as previously reported (12). The *dpb3Δ* replication was slightly slower than the WT strain. The *dpb3Δ mcm2-3A* strain did not display any additive effects on DNA replication progress relative to the *dpb3Δ* strain (Supplementary Figure S5). Of note, the delayed DNA replication progress in the *dpb3Δ* strain may not be related to its defect in a parental histone chaperone, because Dpb3 gene also plays a regulatory role in Pol ϵ DNA extension and DNA repair (45).

**Figure 2.**
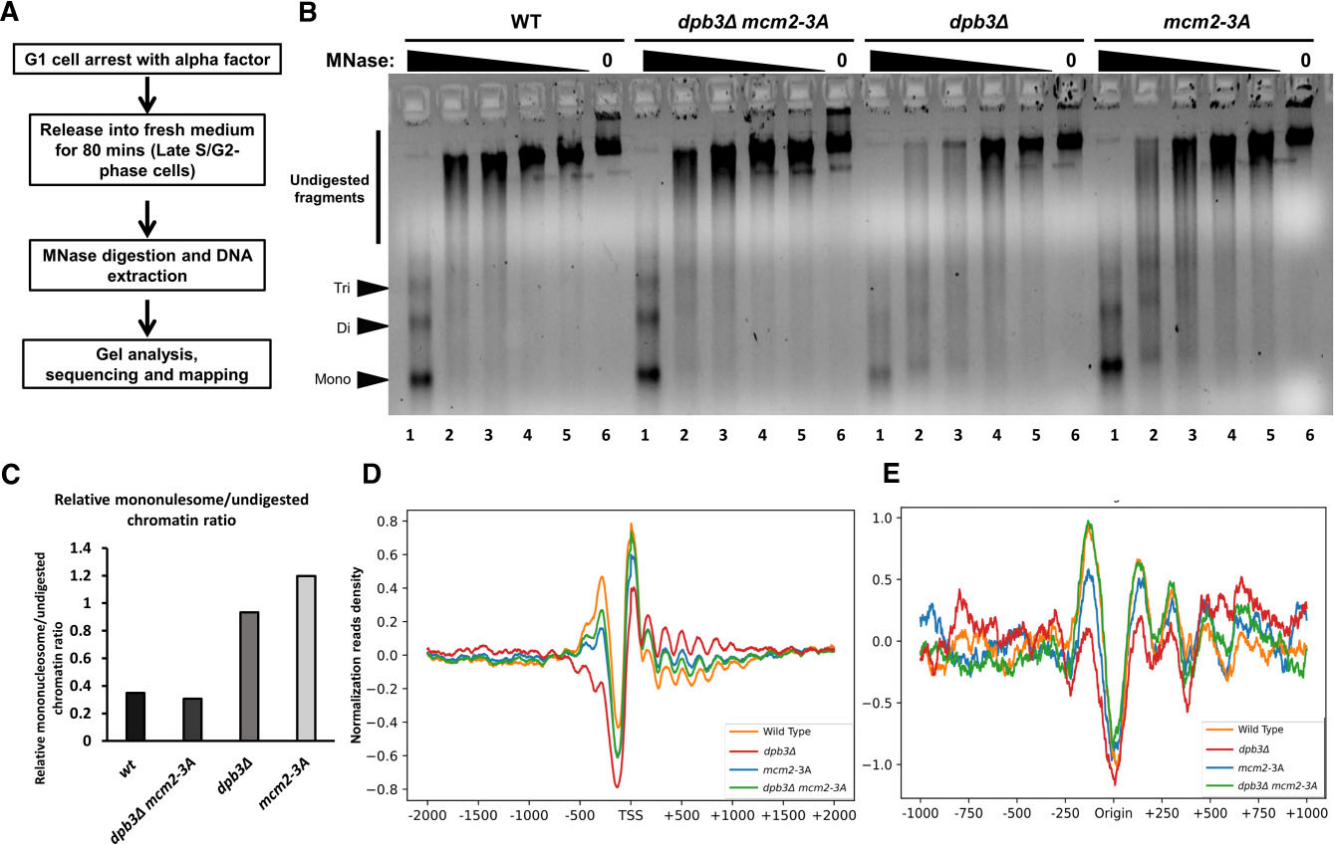
The chromatin-structure changes by *dpb3Δ*, *mcm2-3A* and *dpb3Δ mcm2-3A* mutation at early S phase. (**A**) Procedure used for micrococcal nuclease (MNase) chromatin accessibility assay and MNase-seq, to characterize chromatin accessibility and nucleosome positioning in strains (WT, *dpb3Δ*, *mcm2-3A* and *dpb3Δ mcm2-3A*). Cells were arrested at G1-phase with alpha factor and then released into fresh YPD medium. After 80 min, the samples were collected. The following sample treatment process is described in Materials and method section. (**B**) Results from a micrococcal nuclease (MNase) chromatin accessibility assay for WT, *dpb3Δ*, *mcm2-3A* and *dpb3Δ mcm2-3A* strains, shown on a 2% agarose gel. Chromatin sensitivity assays were performed using digestion with various concentrations of MNase for 10 min followed by quenching with stop solution and DNA extraction. MNase amount from lane 1 to lane 6: 10; 0.5; 0.25; 0.13; 0.06; 0 in Unit. High MNase mount led to smaller fragment and nucleosome bands (poly, tri, di and mononuclesomes) means strong nucleosome positioning. The lane 2 DNA was used for mononulclesome/undigested chromatin fragment calculation in (C). The Lane 1 DNA was used for sequencing library preparation and sequencing data analysis in (D) and (E). (**C**) Calculated relative ratio of mononulclesome/undigested chromatin fragment fraction. The ImageJ software was used to quantify the band intensity. (**D**) MNase-seq profiles of mean nucleosome occupancy around transcription start sites (TSS) for the WT, *dpb3Δ*, *mcm2-3A* and *dpb3Δ mcm2-3A* strains. The y-axis is standardized read counts. Read coverage was calculated at each nucleosome position using reads of length 149–170 bp. For each region surrounding the origins of replication (D) and transcription start sites (**E**), the coverage of the nucleosome was collected and standardized to a mean of 0 and unit variance. Read counts at each position were plotted and can be used to infer nucleosome positioning and relative binding. (**E**) MNase-seq profiles of mean nucleosome occupancy around early replication origin sites for the WT, *dpb3Δ*, *mcm2-3A* and *dpb3Δ mcm2-3A* strains. A complete independent repeat was provided in Supplementary Figure S6.

To explore whether parental histone chaperone mutants display chromatin structural changes, we performed a micrococcal nuclease (MNase) sensitivity assay on cellular bulk chromatin. To detect the potential maximum chromatin structure alteration due to replication-coupled chromatin assembly, we used the late S/G2 phase cells, which had just finished chromatin replication (Figure 2A). As shown in Figure 2B (lane 2) and Supplementary Figure S6A (lane 4), the *dpb3Δ* and *mcm2-3A* mutants are more sensitive to the MNase than the WT or *dpb3Δ mcm2-3A* as visualized by the higher ratio of mononucleosome to large undigested fragments (Figure 2C and Supplementary Figure S6B). To further validate the gel digestion result, we carried out MNase-seq to characterize the nucleosome positioning defect in the mutants. For this analysis, DNA in lane 1 (Figure 2B and Supplementary Figure S6A), composed by the majority of mononucleosome DNA, was used to construct a DNA sequencing library. Subsequently, after library preparation and sequencing, the reads were mapped to the yeast genome. The average nucleosome position profile of all genes with transcription starts sites (TSS) revealed a nucleosome-free region (NFR) and well-positioned nucleosomes (+1 to +6 nucleosomes) surrounding the TSS (Figure 2D and Supplementary Figure S6C). The nucleosome positions in all mutant strains were not noticeably different from those in the WT strain. However, the *dpb3Δ* and *mcm2-3A* strains displayed less clear positioning at the +1 nucleosomes relative to the WT and *dpb3Δ mcm2-3A* strain (Figure 2D and Supplementary Figure S6C). MNase-seq data were also analyzed and mapped to replication origins, and an NFR was also clearly observed there (Figure 2E and Supplementary Figure S6D). Similar to the TSS regions, nucleosome positioning and occupancy at the origins of replication were also weaker in *dpb3Δ* and *mcm2-3A* mutants than in the WT or *dpb3Δ mcm2-3A* strains (Figure 2E and Supplementary Figure S6D). These results suggest that altered parental histone transfer in *dpb3Δ* or *mcm2-3A* mutants results in changes in chromatin structure, and the *dpb3Δ mcm2-3A* double mutant appears to restore the chromatin structure to a state resembling the WT, as opposed to the *dpb3Δ* or *mcm2-3A* single mutants. In conclusion, asymmetric parental histone transfer appears to affect chromatin structure, and the symmetric parental histone deposition in the *dpb3Δ mcm2-3A* double mutant seems to restore the chromatin structure to a state similar to WT.

To investigate whether the chromatin structure changes in the *dpb3Δ* or *mcm2-3A* mutant result in global changes to the levels of histone tail modifications or the major chromatin silencer protein Sir2, we performed western blot analysis on whole-cell protein lysates with growth-phase cells (Supplementary Figure S7A). Levels of H3K36me3, which are associated with active gene transcription (46), were slightly lower in the *dpb3Δ*, *mcm2-3A* and *dpb3Δ mcm2-3A* mutants than in the WT strain (Supplementary Figure S7A, B). This indicates that the parental histone H3–H4 tetramer transfer defect in *dpb3Δ*, *mcm2-3A* and *dpb3Δ mcm2-3A* mutants may lead to reduced H3K36me3 in replicating cells. However, we observed no noticeable differences between the *dpb3Δ*, *mcm2-3A*, *dpb3Δ mcm2-3A* and WT strains with regard to H3K56ac, H3K4me3 or Sir2 levels suggesting that defect in parental histone transfer have minimal impact on global histone tail modifications and Sir2 level.

### Deficiency in parental histone chaperones increases genome instability

As we observed that chromatin structure is altered in *dpb3Δ* and *mcm2-3A* mutants, we also tested their sensitivity to replication stress by exposing them to hydroxyurea. The *dpb3Δ*, *mcm2-3A* and *dpb3Δ mcm2-3A* double mutant did not show any growth defect on 50 mM hydroxyurea (HU) plate (Supplementary Figure S7C), but the *dpb3Δ mcm2-3A* double mutant is a slightly sensitive to 200 mM hydroxyurea (later in Figure 6F).

**Figure 3.**
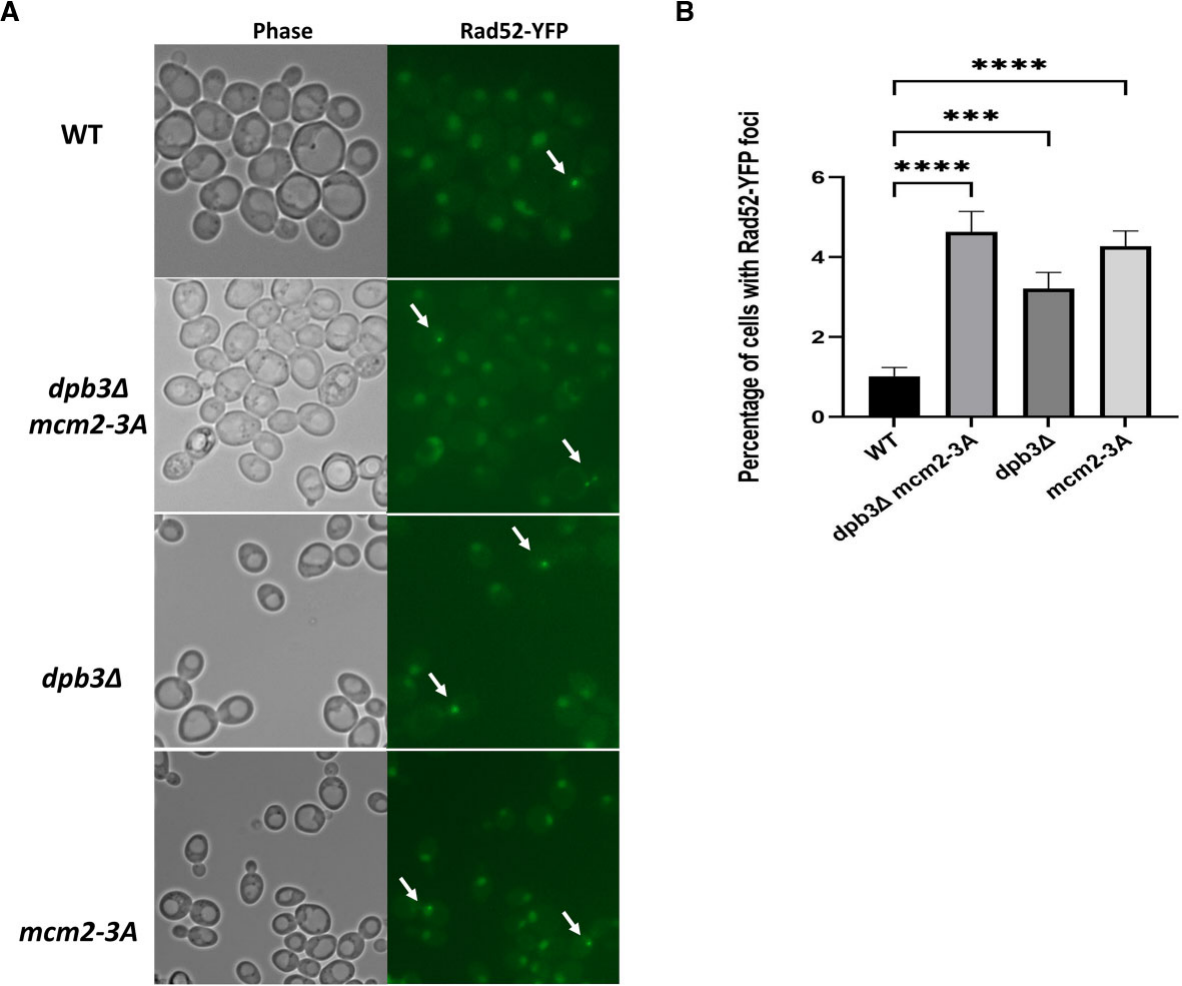
Parental histone chaperone mutants exhibit a slight increase in DNA lesions during S-phase. (**A**) Representative fluorescence images of Rad52-YFP expression, which represent sites of DNA lesion repair, in WT, *dpb3Δ*, *mcm2-3A* and *dpb3Δ mcm2-3A* strains. The Rad52-YFP foci are marked with white arrows. (**B**) Average percentage of cells with Rad52-YFP foci in WT, *dpb3Δ*, *mcm2-3A* and *dpb3Δ mcm2-3A* strains. A one-way ANOVA analysis was used for comparing WT and each mutant. Asterisks indicate statistical significance between two strains. **P < 0.01, ***P  < 0.001, ****P  < 0.0001. Error bars depict standard error of the mean. Over 500 cells were counted.

Next, to investigate whether the parental histone chaperone mutants displayed an elevated frequency of DNA strand breaks, we used fluorescence microscopy to measure the occurrence of Rad52 foci, which serve as markers for DNA lesion repair (39). The number of Rad52 foci in the *dpb3Δ*, *mcm2-3A* and *dpb3Δ mcm2-3A* mutants were nearly twice that of WT cells (Figure 3A, B). These findings suggest an increase in spontaneous DNA strand breaks in parental histone chaperone mutants, even though the Rad52 foci frequency in these mutants is considerably lower than in other mutants with more pronounced Rad52 foci associated with DNA repair deficiencies (47). We acknowledge that a previous study did not report any differences between WT and *mcm2-3A* mutants in terms of Rad52 foci (12). This could be attributed to factors such as a smaller cell count for analysis (we examined over 500 cells) or subtle differences in strain backgrounds. We also measure the Rad52 foci frequency of new histone chaperone mutant (*cac1Δ, asf1Δ* and *rtt106Δ*). The *asf1Δ* mutants gives much higher Rad52 foci; *cac1Δ* shows a significant higher Rad52 foci frequency; and *rtt106Δ* gives a slightly, but not significant higher frequency (Supplementary Figure S8A). These results are also consistent with previous observations (48–50). Furthermore, we test the genetic interaction between the parental histone chaperones (*dpb3Δ* and *mcm2-3A*) and new histone chaperones (*cac1Δ, asf1Δ* and *rtt106Δ*) in relation to Rad52 foci formation. While the *asf1Δ* gives a much higher percentage of Rad52 foci formation, any combinations (*asf1Δ mcm2-3A dpb3Δ, asf1Δ dpb3Δ, asf1Δ mcm2-3A*) did not give any significant changes comparing *asf1Δ* single deletion (Supplementary Figure S8C). In regard to the *cac1Δ*, the *dpb3Δ* complement the Rad52 foci frequency of *cac1Δ* as the *cac1Δ mcm2-3A dpb3Δ* and *cac1Δ dpb3Δ* give less Rad52 foci (Supplementary Figure S8D). This result seems to be consistent with a recent report that parental histone transfer pathway (*dpb3Δ*) regulates DNA repair pathway choice (26). In the context of *rtt106Δ*, there was a trend toward an additive effect, as *rtt106Δ/mcm2-3A/dpb3Δ* exhibited a higher Rad52 foci frequency compared to the *rtt106Δ* single mutant (Supplementary Figure S8E). We also examined a histone H3–H4 gene deletion mutant (*hht2-hhf2Δ*, composed primarily of histone H3–H4 gene transcripts during S phase (51,52)) and did not observe any significant change compared to WT. When we combined *hht2-hhf2Δ* with *dpb3Δ* and *mcm2-3A* single mutant, we observed a relative higher Rad52 foci frequency (Supplementary Figure S8B). All these findings imply that there are intricate interactions between parental histone transfer and new histone chaperones, and that these interactions likely result from a variety of mechanisms.

**Figure 4.**
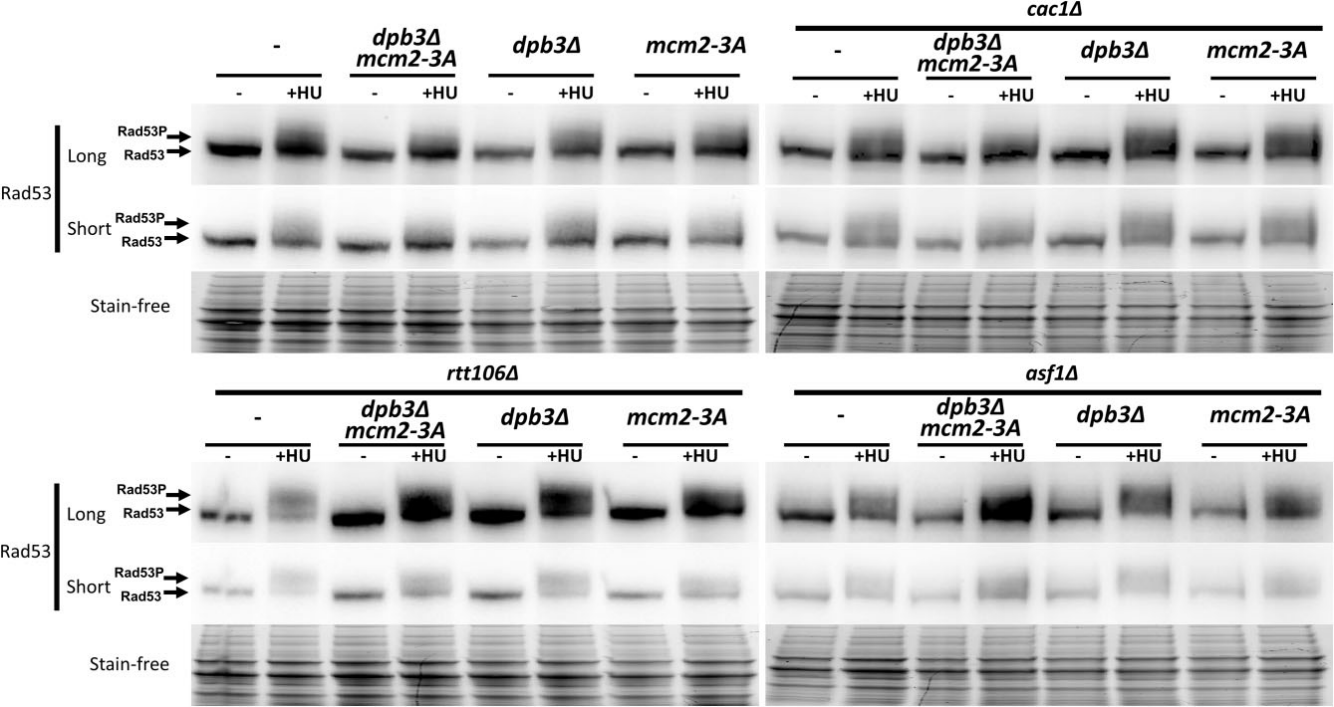
Parental histone chaperone mutants do not show any additional checkpoint kinase Rad53 activation when combining with new histone chaperones under normal growth condition. Immunoblot analysis of Rad53 phosphorylation levels, a marker of the DNA damage response in yeast, before and after treatment with hydroxyurea (HU; a replication fork block agent in different combination of *dpb3Δ, mcm2-3A, dpb3Δ mcm2-3A* with *asf1Δ, cac1 Δ* and *rtt106Δ*. The whole cell lysate with log-phase cells without (left panel) or with 200 mM-HU-treated (1 h) (right panel) were used for these assays. Rad53 antibody (ab104232, Abcam) was used for Rad53 detection. Hyperphosphorylated forms of Rad53 (Rad53-P) will migrate slower in gel analysis. All HU-treated samples give the Rad53-P band compared with untreated samples.

In yeast, DNA strand breaks may activate cell cycle checkpoints. The key checkpoint kinase Rad53 is phosphorylated under DNA damage or replication stress conditions. To examine the Rad53 phosphorylation levels in parental histone transfer mutant cells, we first validated our detection system, including the Rad53 antibody. We successfully detected a distinct Rad53 band under normal culture conditions and the shifted Rad53-P band following hydroxyurea treatment (Supplementary Figure S9A). Next, we tested the parental histone transfer mutants and the combined parental and new histone chaperone mutants (Figure 4). Under normal culture condition, no Rad53-P shifted band was observed in WT, *dpb3Δ*, *mcm2-3A*, or *dpb3Δ mcm2-3A*. *asf1Δ* mutant gives clear Rad53-P band shift and any combination of parental histone chaperone with *asf1Δ* shows a similar band pattern with single *asf1Δ* single mutant. In the case of *cac1Δ* and *rtt106Δ* single mutants and their combination with parental histone chaperone mutants, no Rad53-P band can be detected under normal culture condition. Under HU treatment condition, all tested strain shows Rad53-P activation (Figure 4). In addition, we measured H2A phosphorylation after exposing the strains to methyl methanesulfonate (MMS), a DNA-damaging agent known to induce cellular H2A phosphorylation (53), to verify the effectiveness of our H2A phosphorylation antibody. As expected, MMS significantly increased H2A phosphorylation level although no significant difference observed between WT and parental histone chaperone mutants (Supplementary Figure S9B). All Rad53-P and H2AX data suggest that parental histone chaperone mutations result in increased DNA strand lesions but do not trigger a systemic DNA damage response.

### Spontaneous homologous recombination decreases in parental histone chaperone mutants

**Figure 5.**
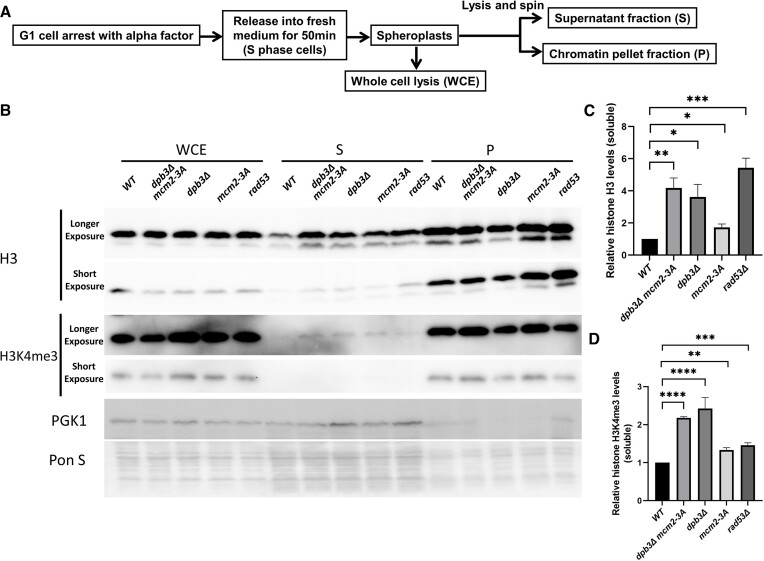
Parental histone chaperone mutations increase free histone levels in the cell. (**A**) Procedure to monitor free histone levels inside WT, *dpb3Δ*, *mcm2-3A* and *dpb3Δ mcm2-3A* strains. (**B**) Immunoblot of H3 and H3K4me3 (a marker for chromatin-associated proteins) and PGK1 (a marker for soluble proteins) in the whole cell extract, soluble fraction, and chromatin fraction of WT, *dpb3Δ, mcm2-3A, dpb3Δ mcm2-3A* and *rad53Δ* strains. H3K4me3 (ab8580 Abcam); PGK1(ab113687 Abcam) and H3-HA (12CA5 Sigma) were used for western blot. We did not observe any obvious H3 and H3K4me3 level difference in whole cell extract. (**C**) Quantitation of soluble H3. The data shown in (C) comes from four independent experiments. Error bars depict standard error of the mean. The signals obtained for soluble histones were normalized to the signals obtained for soluble PGK1 on western blots. Error bars depict standard error of the mean. A one-way ANOVA analysis was used for comparing between two strains. *P  < 0.05, **P  < 0.01, ***P  < 0.001. (**D**) Quantitation of soluble H3K4me3. The data shown in (D) comes from three independent experiments. Error bars depict standard error of the mean. A one-way ANOVA analysis was used for comparing between two strains.

We hypothesized that histones in H3–H4 tetramers that dissociate from DNA in the parental histone chaperone mutants are released as soluble (free) proteins. To test this possibility, we analyzed the amount of non-chromatin-associated H3 during late S-phase (Figure 5A). As a positive control, we used a *rad53Δ* mutant, which is defective in regulating the histone degradation pathway and gives a higher soluble histone level (38,52). The histone H3K4me3 (chromatin fraction marker) and PGK1(cytosol fraction marker) are used as indicators of successful fractionations. In line with the previous report, we detected a higher soluble H3 in the *rad53Δ* mutant (38) (Figure 5B). Notably, a significant increase in soluble H3 was observed in *dpb3Δ*, *mcm2-3A* and *dpb3Δ mcm2-3A* mutants relative to WT cells (Figure 5B, C). Moreover, we also observed higher levels of H3K4me3 in the soluble fraction of *dpb3Δ*, *mcm2-3A* and *dpb3Δ mcm2-3A* mutants than in WT cells (Figure 5B, D). As the *dpb3Δ* and *dpb3Δ mcm2-3A* mutant grow slower than WT, we tested whether the growth rate could influence the level of free histones by taking two additional late S/G2 phase timepoint samples (60 and 80 min). The results suggest that the level of free histones remained constantly higher in the *dpb3Δ, mcm2-3A, mcm2-3A dpb3Δ* and *rad53Δ* mutants compared to WT (Supplementary Figure S10A–F). Also, the *dpb3Δ mcm2-3A* double mutant has a higher soluble histone than the single *dpb3Δ or mcm2-3A* mutant (Figure 5C, D and Supplementary Figure S10A–F). In the next step, we tested whether the observed increased free histone level phenotype can be rescued by overexpressing of new histone chaperones in *dpb3Δ mcm2-3A* mutant. We did not observe any significant changes in the level of free histone upon overexpression of any of Asf1, Rtt106 and Caf1 when compared to the control (empty vector only, Supplementary Figure S11A, B).

**Figure 6.**
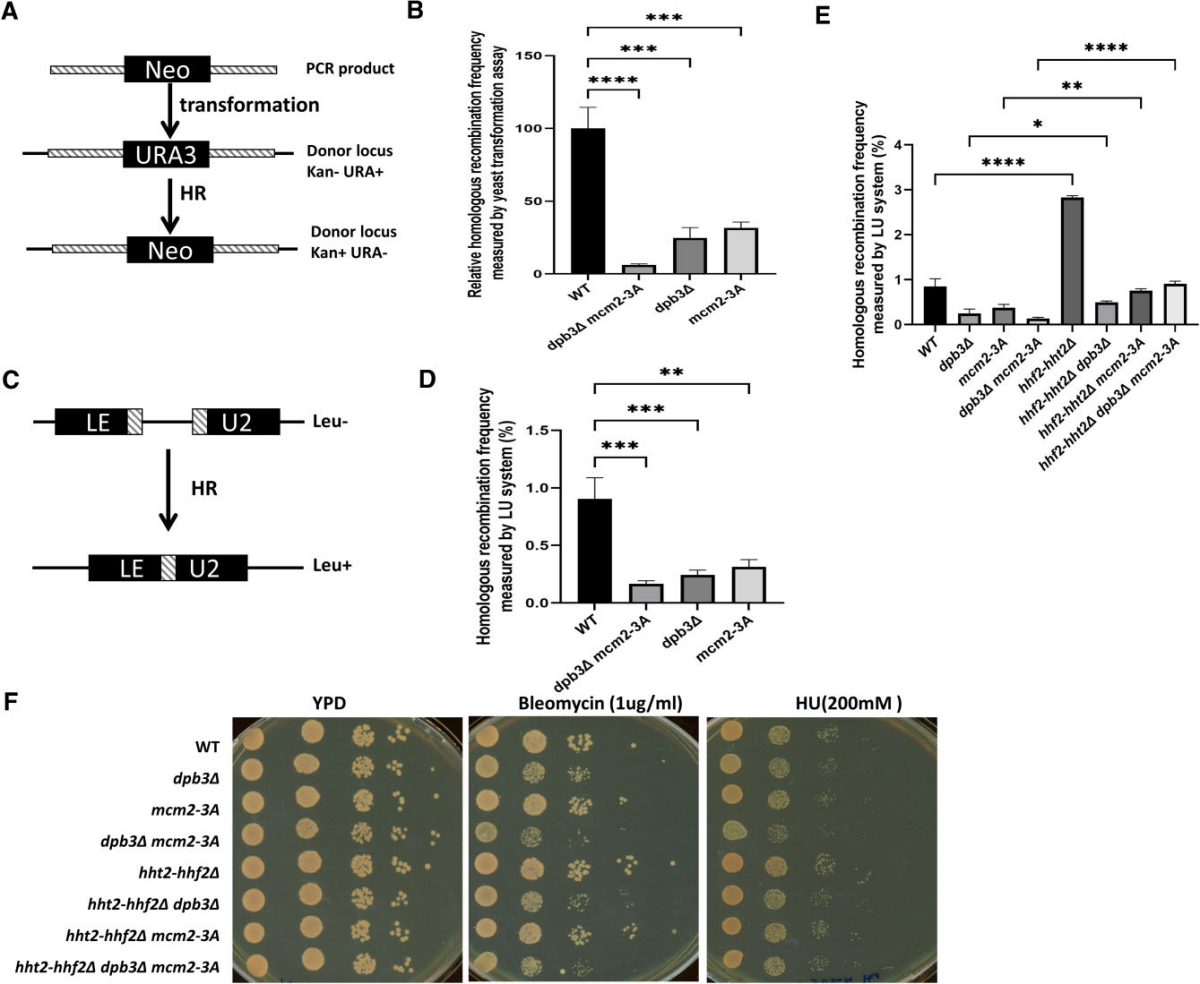
Parental histone chaperone mutations decrease the frequency of homologous recombination. (**A**) Procedure for quantifying HR efficiency in WT, *dpb3Δ*, *mcm2-3A* and *dpb3Δ mcm2-3A* strains transformed with the *Neo* gene flanked by the 750-bp 5′ UTR and 550-bp 3′ UTR of the *URA3* gene. (**B**) Relative HR frequency of *dpb3Δ*, *mcm2-3A* and *dpb3Δ mcm2-3A* mutants to the WT strain. (**C**) Procedure for quantifying HR efficiency using the LU system in different strains. (**D**) Percentage of WT, *dpb3Δ*, *mcm2-3A*, and *dpb3Δ mcm2-3A* cells undergoing HR, as detected using the LU system. (**E**) Deletion of *hht2-hhf2* elevates HR rates of parental histone transfer defective mutants. (**F**) Deletion of *hht2-hhf2* promotes the resistance of *dpb3Δ mcm2-3A* to bleomycin and high concentration of hydroxyurea. In this figure, a one-way ANOVA analysis was used for comparing two different mutants. *P <0.05, **P  < 0.01, ***P  < 0.001, ****P <0.0001. Error bars depict standard error of the mean in this figure.

According to a previous study, removing one of the two copies of the histone H3–H4 gene boosts the HR rate by promoting HR factors to bind to DNA strand breaks (52). Based on their data and the higher levels of soluble histones observed in the *dpb3Δ*, *mcm2-3A* and *dpb3Δ mcm2-3A* mutants, we hypothesized that the mutants might display a decreased HR rate. To test this hypothesis, we performed a direct yeast transformation assay in which HR could be measured by neomycin and fluoroorotic acid (FOA) resistance (Figure 6A). The *dpb3Δ* and *mcm2-3A* mutants showed a nearly 70% reduction in HR compared to the WT strain, and the *dpb3Δ mcm2-3A* double mutant showed <10% the HR frequency of the WT strain (Figure 6B). We did not recover any resistant colonies in the *rad52Δ* mutant used as a negative control (data not shown).

To exclude the possibility that these results could be attributed to variation in the amount of DNA transformed into the cells, we quantified HR using an *in vivo* local homologous recombination assay system (LU), in which HR frequency is indicated by a functional Leu2 gene recovery (Figure 6C) (41). Using this system, we observed a significant reduction in HR frequency in the *dpb3Δ* (∼66%), *mcm2-3A* (59%) and *dpb3Δ mcm2-3A* (∼74%) mutants (Figure 6D).

### Decreased HR frequency resulting from defective parental histone transfer can be rescued by *hhf2–hht2* deletion

Consistent with a previous report (52), we observed that removing one of the two copies of the histone H3–H4 gene (Hhf2–Hht2) boosts the HR rate. Furthermore, we observed that the *hhf2-hht2Δ* can partially complement the HR rates of *dpb3Δ, mcm2-3A* and *dpb3Δ mcm2-3A* (Figure 6E). As the dosage of histone genes regulates the sensitivity of budding yeast cells to DNA-damaging agents, we assessed their sensitivity to such agents (Bleomycin and hydroxyurea). The *dpb3Δ mcm2-3A* shows slightly sensitivity to 1 ug/ml bleomycin or 200 mM HU, but *hhf2-hht2Δ* partially complement this sensitivity (Figure 6F and Supplementary Figure S12A, B). These results further support the parental histone transfer mutants regulate the HR frequency through histone dosage control.

### Deletion of histone chaperone Asf1 increases the HR frequency of *dpb3Δ mcm2-3A* mutant.

Cac1 and Asf1 were shown to be involved in repair of double strand break through HR pathway (24,25,54). Next, we tested whether the overexpression of new histone chaperones (Caf1 or Asf1 or Rtt106) can restore the decreased HR frequency in *dpb3Δ mcm2-3A* mutant. We examined the effect of overexpression of Caf1, Rtt106 and Asf1 on HR frequency in both WT and parental histone transfer mutant (*dpb3Δ mcm2-3A*) (44). In comparison to vector control transformants, we did not observe an increase in HR frequency with the overexpression of Caf1, Asf1, or Rtt106 (Supplementary Figure S11C, D). Interestingly, overexpression of Caf1 in WT or *dpb3Δ mcm2-3A* resulted in substantial decrease in HR frequency compared to their own controls (Supplementary Figure S11C, D). Overexpression of Rtt106 significantly decreases HR frequency only in the *dpb3Δ mcm2-3A* double mutant.

In addition, we also assessed the impact of deletions of these new histone chaperones on HR frequency in *dpb3Δ mcm2-3A* mutant. Consistent with a previous report, the deletion of Cac1 or Rtt106 did not alter the HR frequency compared to WT, but the deletion of Asf1 significantly increased the HR frequency (48) (Supplementary Figure S11E). When introducing the new histone chaperone deletion mutations into *dpb3Δ mcm2-3A* background, the HR frequency of *dpb3Δ mcm2-3A rtt106Δ*, and *dpb3Δ mcm2-3A cac1Δ* was similar to that of *dpb3Δ mcm2-3A*, while *dpb3Δ mcm2-3A asf1Δ* showed a significantly higher HR frequency than *dpb3Δ mcm2-3A* mutant (Supplementary Figure S11E). These results, along with our Rad52-YFP foci counting data (Supplementary Figure S8) support a former model that *asf1Δ* generate more double strand breaks, thereby increasing HR (54). In conclusion, defective parental histone pathways cannot be rescued by supplying more new histone chaperones and Caf1 overexpression is detrimental to innate cellular DNA repair pathways. *asf1Δ* does increase the HR frequency of *dpb3Δ mcm2-3A*, but the mechanism is likely due to more DSB generated.

### Genetic interaction between FACT and Mcm2 or Dpb3

FACT (facilitates chromatin transcription) has been implicated in nucleosome regulation (12,55–58). Recent studies have expanded this understanding, revealing FACT’s involvement in both leading and lagging strand parental histone transfer processes (14). Building on these findings, we investigated the genetic interaction between FACT and the two parental histone pathway mutants concerning cell growth and cellular homologous recombination frequency. FACT comprises two subunits, Spt16 and Pob3 (59). To explore these interactions, we utilized the temperature-sensitive mutant *spt16-11* (60), which is lethal at 37°C. Interestingly, *spt16-11* exhibited a synthetic growth defect, sensitivity to Bleomycin with *dpb3Δ* single mutants or *mcm2-3A dpb3Δ* double mutants (Supplementary Figure S12C). However, the growth rate of the *spt16-11 mcm2-3A* double mutant was comparable to that of the *spt16-11* single mutant (Supplementary Figure S12C). In terms of homologous recombination frequency, the *spt16-11* mutant displayed a significant increase in HR frequencies. This result is expect considering significant higher free histone in Spt16 mutant (58). Surprisingly, combining *spt16-11* with *mcm2-3a*, *dpb3Δ* or *mcm2-3a dpb3Δ* did not further decrease the HR frequency (Supplementary Figure S12D). Given FACT’s known involvement in both general transcriptional and replication regulation (55–57,59), additional studies will be essential to determine the direct relevance of these genetic interactions to FACT’s function in parental histone transfer.

## Discussion

**Figure 7.**
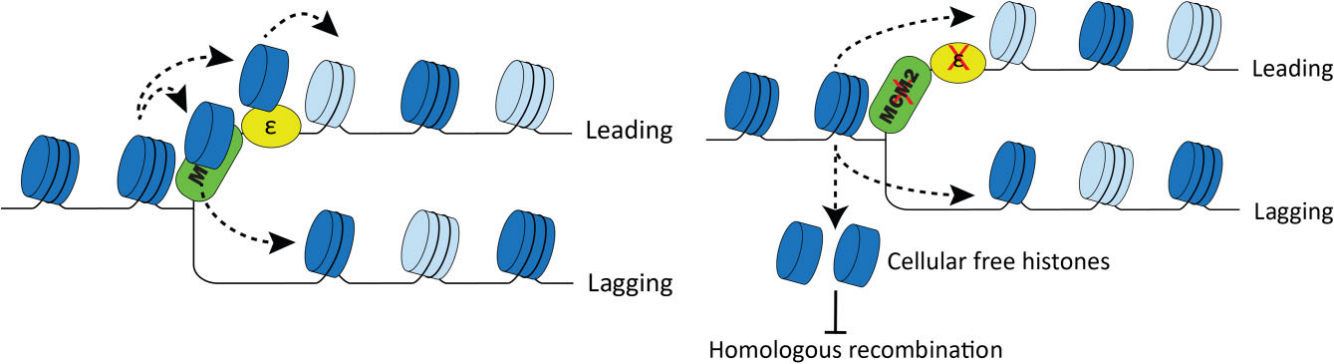
A proposed model showing that defective parental histone transfer impairs homologous recombination. In the wild type cells (left panel), parental histone H3–H4 (dark blue) can be efficiently transfer to newly synthesized DNA mediated through Polymerase ϵ subunit Dpb3/Dpb4 and Mcm2-Ctf4-Polα axis following DNA replication fork. The new histone H3–H4 (light blue) is deposited following the empty space left by the parental histone. In the *dpb3Δ mcm2-3A* mutant (right panel), a small fraction of parental histone H3–H4 may leave chromatin and release into free H3–H4. As a consequence, the higher free histone level may inhibit HR through inhibiting ssDNA resections and host HR factors (52).

The purpose of this study was to elucidate the role of the two parental histone transfer pathways by comparing a yeast strain with mutations in both Dpb3 and Mcm2 to single Dpb3 and Mcm2 mutants, as well as the WT strain. We performed a variety of experiments to determine the effect of disrupting each pathway upon the strand bias of parental histone transfer, chromatin structure, genomic instability, and HR. Our results collectively support the model that mutations in parental histone H3–H4 chaperones reduce HR frequency, likely due to the elevated levels of free histones (Figure 7). Our findings consistently showed that the proper transfer of parental histones to the leading and lagging strands of DNA during replication is required to maintain chromatin structure and genome integrity.

### Parental histone transfer and epigenetic memory

During cell division, epigenetic patterns, including histone tail modifications, must be copied to maintain proper gene expression. DNA sequence–dependent mechanisms apparently play a role in the duplication of histone tail modifications. For example, a silencer DNA sequence is located at the silenced mating type locus in budding yeast. When the silencer sequence is deleted, the silenced gene is expressed after cell division (61). In addition to the sequence-dependent gene silencing mechanism, a ‘copy and paste’ mechanism has been proposed to explain epigenetic inheritance (62). The PRC2 complex, a major gene silencer, can bind to the histone tail modification H3K27me3 and promote further H3K27me3 modification locally (63). Similarly, in the absence of demethylase, Clr4 can perform this reader-writer function to copy H3K9me3 modifications in fission yeast (62).

In budding yeast, the major mechanism responsible for gene silencing involves histone deacetylases, such as Sir2 and others sir proteins (61). In our study, the *mcm2-3A* and *dpb3Δ* parental histone chaperone mutants showed strong strand bias in parental histone transfer during DNA replication, a striking contrast from the almost total lack of bias in the WT strain. If a ‘copy and paste’ mechanism plays a key role in silencing at the *HML* locus in budding yeast, we would expect to observe a dramatic loss of silencing in these two mutants. However, our CRASH assay results showed the loss of silencing in both mutants was only moderate and much weaker than that in the new histone chaperone Caf1 mutant (*cac1Δ*). Like the *mcm2-3A* and *dpb3Δ* mutants, the *dpb3Δ mcm2-3A* mutant showed little strand bias with regard to parental histone H3–H4 tetramer transfer. However, as reported previously (16), this double mutant showed a stronger loss of silencing than either of the single mutants. Collectively, these data do not support the existence of a copy and paste mechanism in budding yeast. In this study, we only investigated silencing at the *HML* locus; whether our findings hold across the genome, such as in sub-telomeric regions, or in other organisms needs further testing.

### Free histone levels in parental histone chaperone mutants

In this study, we found that the frequency of HR in parental histone chaperone mutants (*mcm2-3A*, *dpb3Δ*) was significantly lower than in the WT strain. The *dpb3Δ mcm2-3A* mutant showed an additive defect. Our biochemical analysis revealed that the underlying mechanism involved high levels of free histones in the mutants, likely released from chromatin. Recently, another research group reported that the *dpb3Δ* mutation can partially rescue the *ctf4Δ* mutant's greater sensitivity to the DNA damaging agent methylmethane sulphonate (MMS) (26). They also observed that the frequency of template-switch copying (HR dependent) was lower in *ctf4Δ* mutant. With the additional *dpb3Δ* mutation, the template-switch was still defective and the frequency of translesion synthesis (HR independent) was higher. These observations are consistent with our findings that parental histone chaperone mutants decrease HR. The same research group showed that *mcm2-3A* and *pol1-2A* deletions, both of which affect parental histone transfer to the lagging DNA strand, were not able to rescue the MMS-sensitivity phenotype of the *ctf4Δ* mutant. This finding is consistent with our previous work (13), which showed that Mcm2, Ctf4, and Pol1 function in the same pathway to mediate parental histone transfer to the lagging strand. Thus, combining these mutations (*mcm2-3A*, *Ctf4Δ*, and *Pol1-2A*) would not be expected to further decrease HR frequency. While our current observations strongly support the free histone model (Figure 7), it is important to acknowledge that other indirect factors, altered by parental histone transfer defects, could potentially influence the homologous recombination (HR) frequency.

It is interesting to know the impact of the mutation of newly synthesized histone H3–H4 chaperones on the frequency of HR. The newly synthesized histone chaperones Caf1 and Asf1 promote HR through nucleosome assembly in human cells (24). In human cell lines, immunofluorescence staining shows that Caf1 is recruited to DNA double strand breaks (21). Paradoxically, in plants, Caf1 mutants show an increased frequency of HR, probably due to greater DNA accessibility (20). Our results in budding yeast show that parental histone chaperones promote HR, but the mechanism responsible appears to involve the level of free histones in the cell rather than nucleosome assembly (Figure 7). A recent *in vitro* chromatin replication study showed that higher levels of free histones decrease the efficiency of parental histone transfer (64). As we observed higher free histone levels in parental histone chaperone mutants, the efficiency of local parental histone transfer would be predicted to fall in these strains as well. The loss of silencing we observed at the *HML* locus in parental histone chaperone mutants is consistent with this prediction although it may directly be due to the defect in parental histone chaperones.

## Supplementary Material

gkae205_Supplemental_File

## Data Availability

The sequencing data reported in this paper is available in the Gene Expression Omnibus database under accession number GSE240331.
