# Supplementary material for: Defective transfer of parental histone decreases frequency of homologous recombination by increasing free histone pools in budding yeast
Source: Nucleic Acids Res. 2024 Mar 30;52(9):5138–51. doi: 10.1093/nar/gkae205 (PMC11109958; doi:10.1093/nar/gkae205)
Supplement: gkae205_Supplemental_File [file gkae205_supplemental_file.pdf]

Defective transfer of parental histone decreases frequency of homologous recombination by increasing free histone pools in budding yeast

Srinivasu Karri[1], Yi Yang[1], Jiaqi Zhou[2], Quinn Dickinson[1], Jing Jia[1], Yuxin Huang[2], Zhiquan Wang[3], Haiyun Gan[2*], and Chuanhe Yu[1*]

[1]Hormel Institute, University of Minnesota, Austin, MN 55912 USA

[2]CAS Key Laboratory of Quantitative Engineering Biology, Guangdong Provincial Key Laboratory of Synthetic Genomics and Shenzhen Key Laboratory of Synthetic Genomics, Shenzhen Institute of Synthetic Biology, Shenzhen Institutes of Advanced Technology, Chinese Academy of Sciences, Shenzhen, 518055, China

[3]Division of Hematology, Department of Medicine, Mayo Clinic, Rochester, MN, 55905 USA

*Corresponding authors:

Dr. Chuanhe Yu
E-mail: yu000479@umn.edu

Dr. Haiyun Gan
hy.gan@siat.ac.cn

Key words: Dpb3, Dpb4, Mcm2, homologous recombination, histone chaperones, eSPAN, chromatin replication

Running title: Efficient parental histone transfer promotes homologous recombination

Supplemental Figure 1. A graphic outline of eSPAN experimental procedure.

The graphic outlines the hypothetical outcomes for the symmetric newly synthesized and parental histone H3-H4 eSPAN peaks, with new and parental $(H3\text{-}H4)_2$ tetramers at two replicating strands. The sample collection procedure was as Fig 1A. The chromatin was digested into the majority of Mono/di-nucleosome with MNase. The digested chromatin was immunoprecipitated with H3K4me3 (parental) or H3K56ac(new) specific antibodies. ChIPed DNA was first denatured and then undergo BrdU-IP by BrdU specific antibody. The eSPAN samples were constructed into the library with a single strand specific method (1). The red and green lines represent the Watson and Crick strands, respectively. In this situation, both parental histone and new histone eSPAN peaks will have no strand bias. Bias calculation at individual nucleosomes (two in the cartoon) is shown at the bottom.

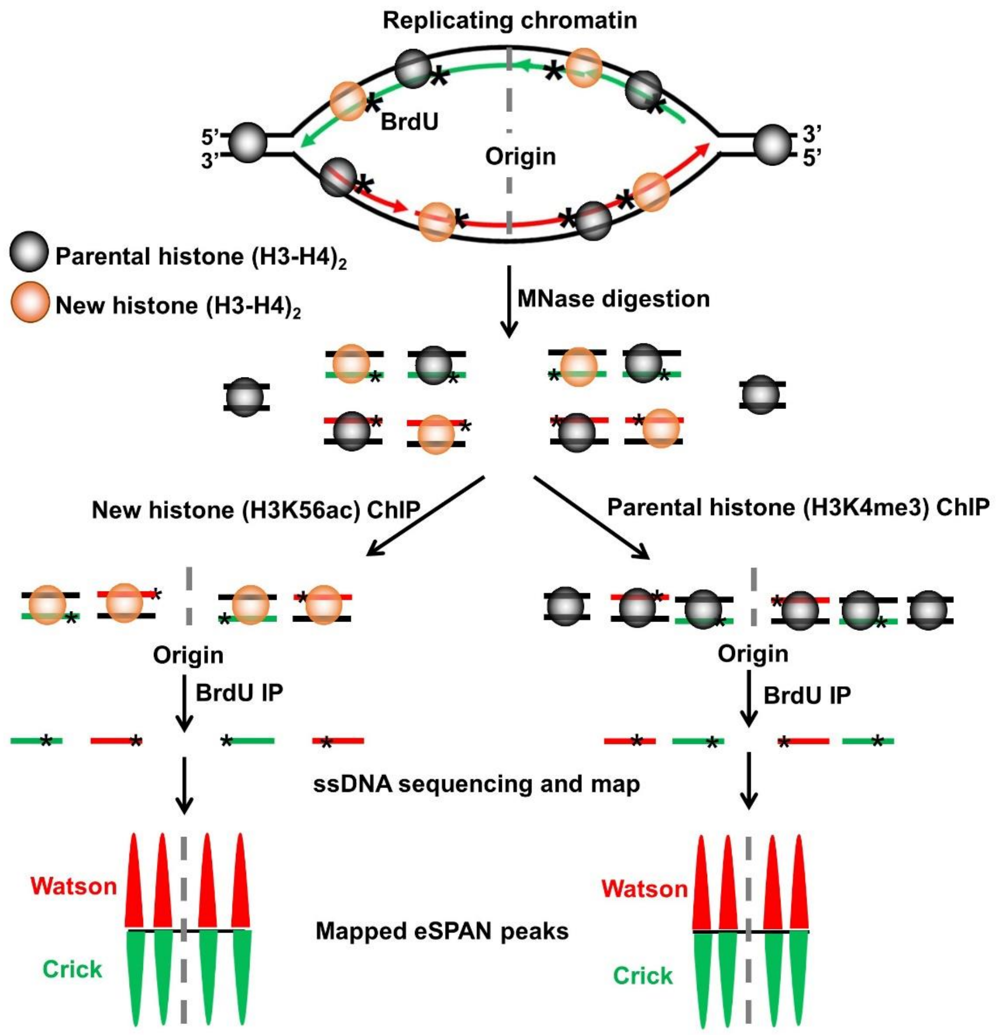

Supplemental Figure 2. Combination of *dpb3Δ* and *mcm2-3A* mutations neutralizes the strand bias of new histone H3-H4 tetramers (H3K56ac) in single *dpb3Δ* or *mcm2-3A* mutants.

(**A**) Snapshot of H3K56ac ESPAN read enrichment at leading and lagging strands at early replication origin ARS1309 in wild-type (WT), *dpb3Δ*, *mcm2-3A*, and *dpb3Δ mcm2-3A* strains. The sequence reads were mapped to both the Watson strand (red) and the Crick strand (green) of the reference genome. The eSPAN experimental procedure following **Fig 1A**. (**B-E**) *Top*: Heatmaps representing the bias ratio of newly synthesized histone H3 (H3K56ac) eSPAN peaks for WT, *dpb3Δ*, *mcm2-3A*, and *dpb3Δ mcm2-3A* strains at each of the 10 nucleosomes surrounding each of the 134 early DNA replication origins. Individual nucleosomes are represented by the circles at the top of the heatmaps, and their positions are indicated relative to the origin (−10 to +10). Each row represents the average log2 Watson/Crick ratio of H3K56ac eSPAN sequence reads at one origin. *Bottom*: Average bias ratio of newly synthesized histone H3 (H3K56ac) eSPAN peaks for WT, *dpb3Δ*, *mcm2-3A*, and *dpb3Δ mcm2-3A* strains at each of the 10 nucleosomes surrounding the 134 early replication origins.

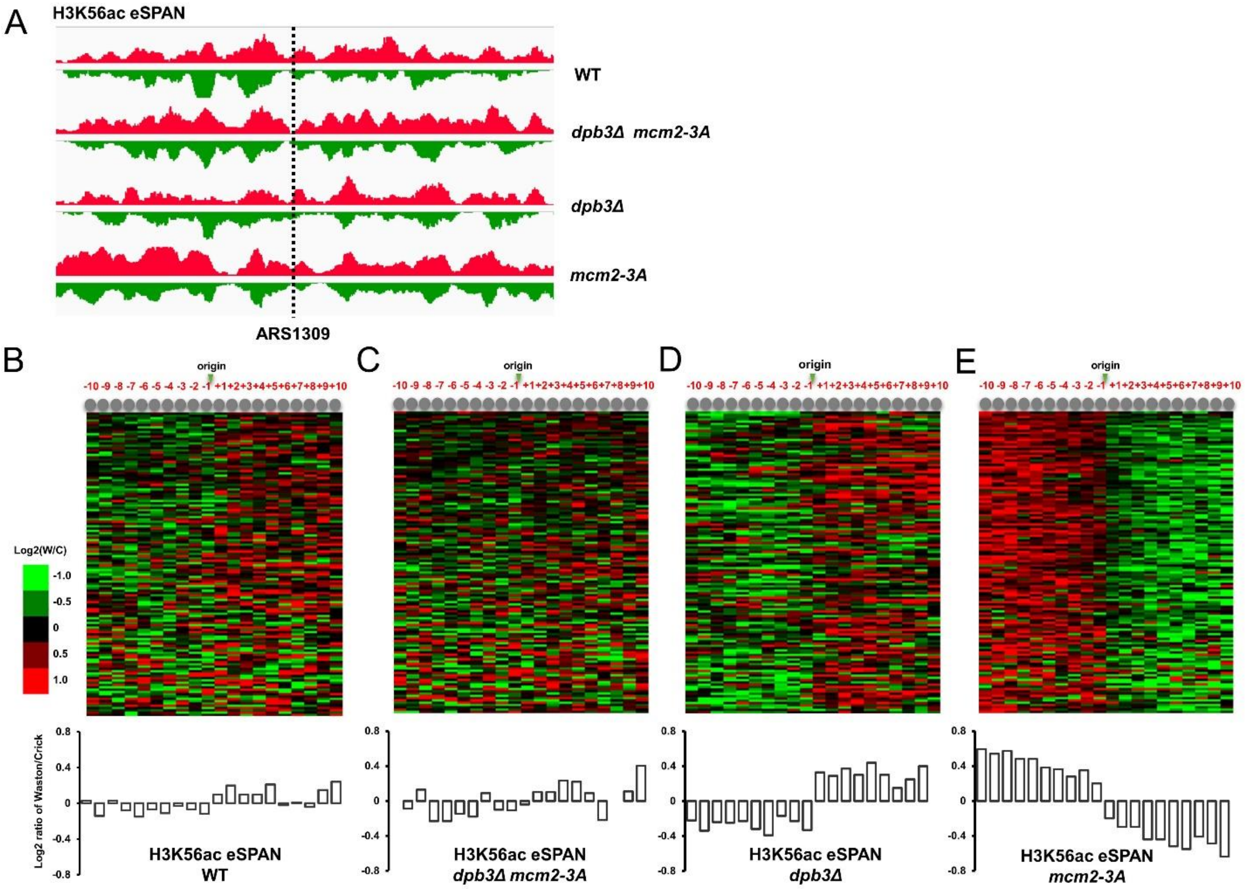

Supplemental Figure 3. New histone chaperone mutations (*cac1Δ, asf1Δ and rtt106Δ*) show little effect on parental histone transfer.

(**A**) Snapshot of H3K4me3 eSPAN read enrichment at leading and lagging strands at early replication origin ARS1309 in WT, *cac1Δ*, asf1*Δ and rtt106Δ* strains. The sequence reads were mapped to both the Watson strand (red) and the Crick strand (green) of the reference genome. The H3K4me3 eSPAN experimental procedure following **Fig 1A**. (**B-D**) *Top*: heatmaps representing the bias ratio of parental histone H3 (H3K4me3) eSPAN peaks for *cac1Δ*, asf1*Δ and rtt106Δ* strains at each of the 10 nucleosomes surrounding each of the 134 early DNA replication origins. Individual nucleosomes are represented by the circles at the top of the heatmaps, and their positions are indicated relative to the origin (−10 to +10). Each row represents the average log2 Watson/Crick ratio of H3K4me3 eSPAN sequence reads at one origin. *Bottom*: average bias ratio of parental histone H3 (H3K4me3) eSPAN peaks for *cac1Δ*, *asf1Δ and rtt106Δ* strains at each of the 10 nucleosomes surrounding the 134 early replication origins.

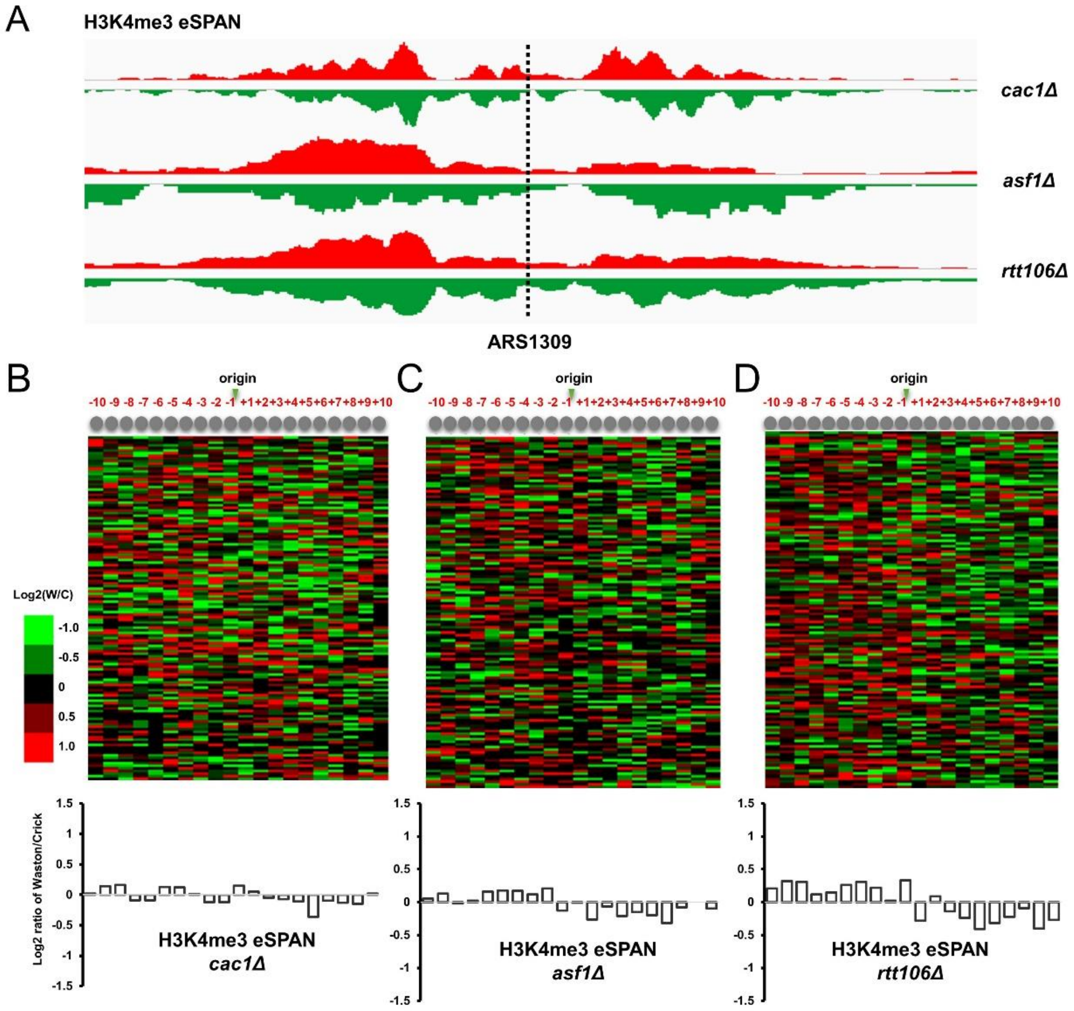

Supplemental Figure 4. Effect of parental histone chaperone mutations on loss of silencing at the *HML* locus, as determined by CRASH assay.

(**A**) Fluorescence images of colonies derived from WT, *dpb3Δ, mcm2-3A, dpb3Δ/mcm2-3A,* and *cac1Δ* strains containing the RFP-GFP cassette at the HMLα::cre locus. The bright green sector in GFP channel or dark sector in RFP channel represent the loss of silence. (**B**) *WT, dpb3Δ, mcm2-3A, dpb3Δ/mcm2-3A,* and *cac1Δ* strains showed different degrees of silencing at the HML locus when analyzed by CRASH assay. A one-way ANOVA analysis was used for comparing between two strains. Error bars depict standard error of the mean. Asterisks indicate statistical significance between two strains. *p < 0.05, **p < 0.01, ***p<0.001, ****p<0.0001.

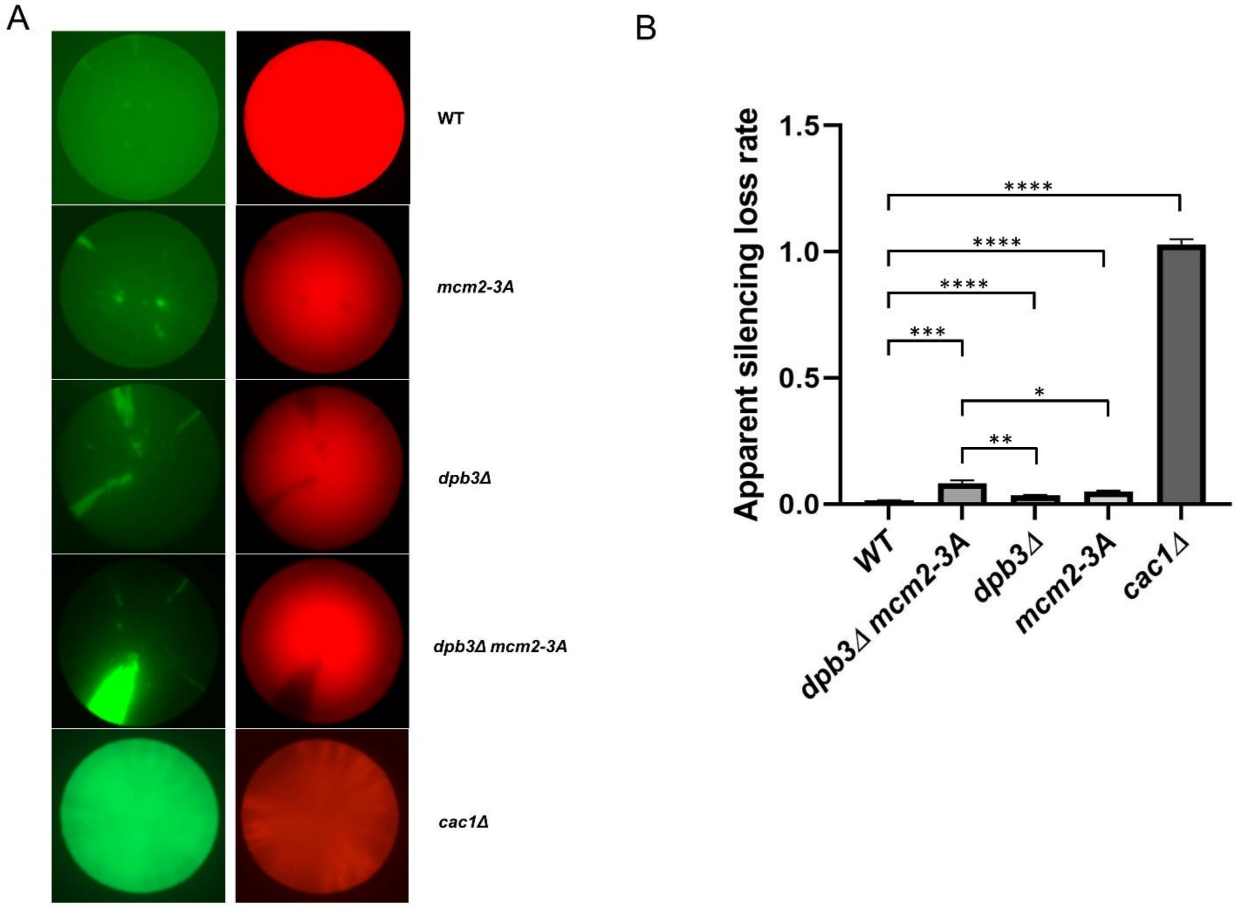

Supplemental Figure 5. Cell-cycle progression in the parental histone chaperone mutants.

Cell-cycle progression of the WT, *dpb3Δ*, *mcm2-3A*, and *dpb3Δ mcm2-3A* strains were monitored by flow cytometry after cells were released from arrest in G1-phase. A mating pheromone was added 60 min after release from G1-phase so that completion of cell division could be monitored by the disappearance of 2C cells and the reappearance of 1C cells.

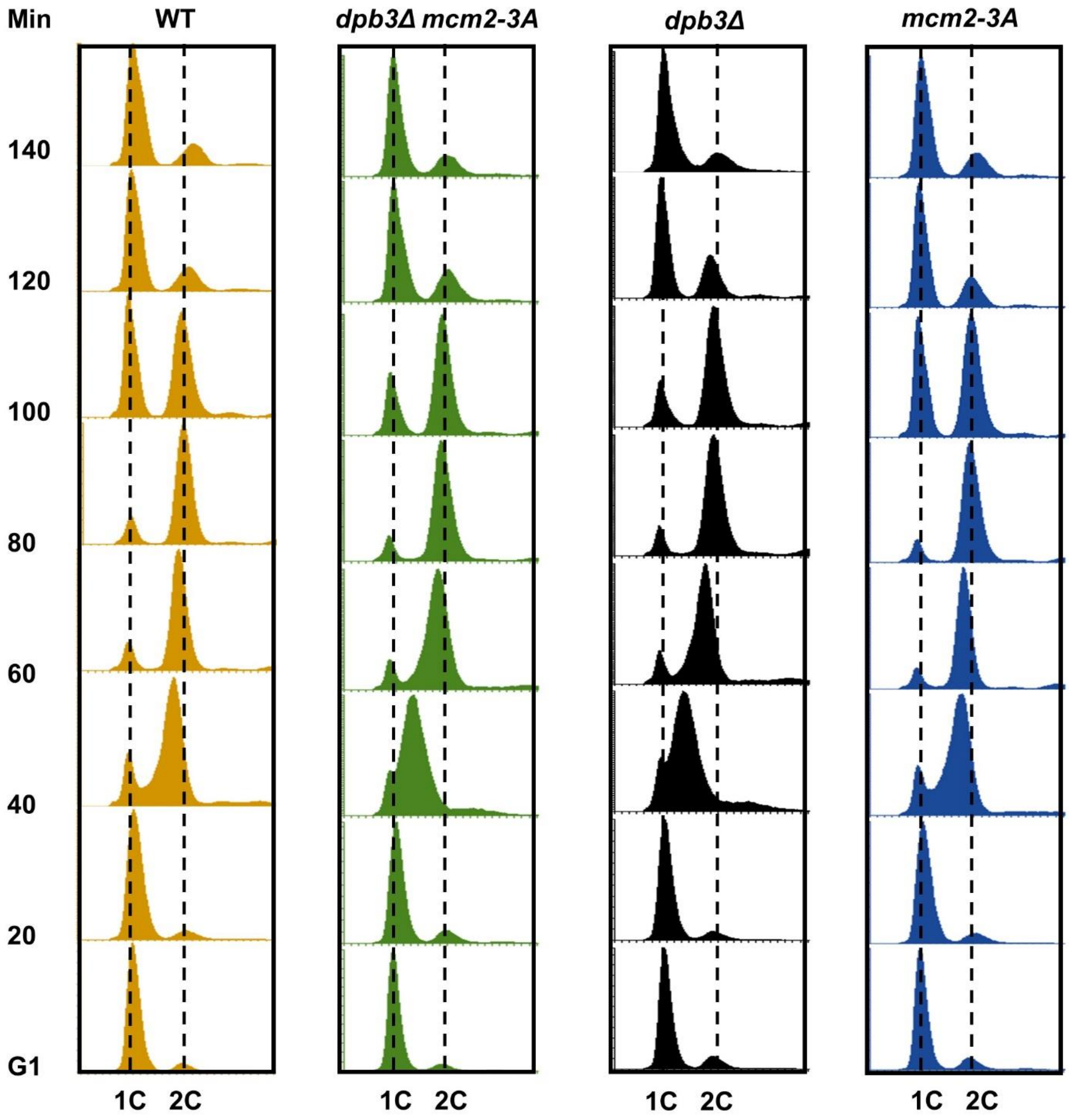

Supplemental Figure 6. Chromatin accessibility assay on WT, *dpb3Δ, mcm2-3A, and dpb3Δ mcm2-3A* mutants at late S phase.

(**A**) Results from a micrococcus nuclease (MNase) chromatin accessibility assay for WT, *dpb3Δ*, *mcm2-3A*, and *dpb3Δ mcm2-3A* strains, shown on a 2% agarose gel. This assay is a repeat of Figure 2 with different MNase amount. Chromatin sensitivity assays were performed using digestion with various concentrations of MNase for 10 min followed by quenching with stop solution and DNA extraction. MNase amount used from lane 1 to lane 6 are: 40;10; 2.5; 0.6; 0.15;0 in Unit. High MNase mount led to smaller fragment and nucleosome bands (poly, tri, di, and mononuclesomes) mean strong nucleosome positioning. The lane 4 DNA was used for mononulclesome/undigested chromatin fragment calculation in (B). The lane 1 DNA was used for sequencing library preparation and sequencing data analysis in (C) and (D). (**B**) Calculated relative ratio of mononulclesome/undigested chromatin fragment fraction. The ImageJ software was used to quantify the band intensity. (**C**) MNase-seq profiles of mean nucleosome occupancy around transcription start sites (TSS) for the WT, *dpb3Δ*, *mcm2-3A*, and *dpb3Δ mcm2-3A* strains. (**D**) MNase-seq profiles of mean nucleosome occupancy around early replication origin sites for the WT, *dpb3Δ*, *mcm2-3A*, and *dpb3Δ mcm2-3A* strains.

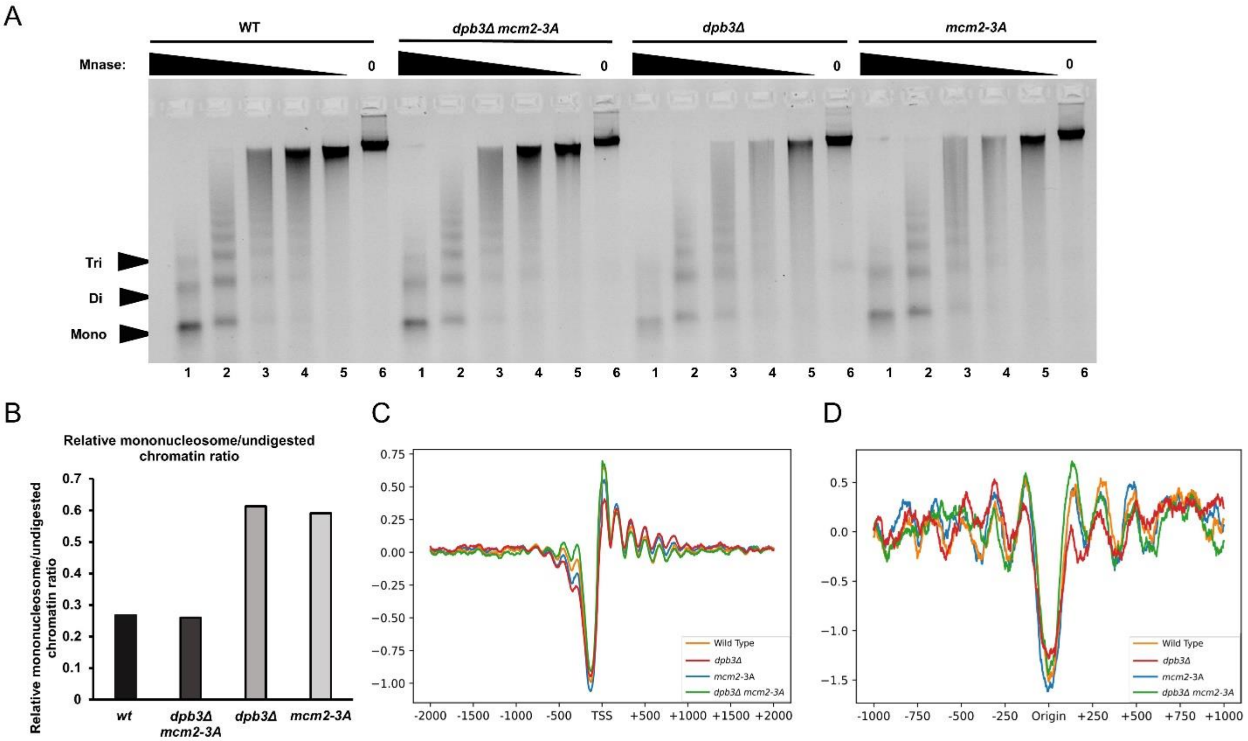

Supplemental Figure 7. Histone modifications and cell growth in the parental histone chaperone mutants.

(**A**) Immunoblot analysis of histone modification levels in whole cell extracts (WCE) from WT, *dpb3Δ*, *mcm2-3A*, and *dpb3Δ mcm2-3A* strains. H3K4me3 (ab8580 Abcam); H3K56ac (2); H3K36me3 (ab9050 Abcam); PGK1(ab113687 Abcam); Sir2 (CS1102,(3)) antibodies were used in these analyses. (B) Calculated relative H3K36me3, H3K56ac, H3K4me3 and Sir2 level. The data shown in comes from three independent experiments. The signals were normalized to the signals obtained for soluble PGK1 on western blots. Error bars depict standard error of the mean. A one-way ANOVA analysis was used for comparing between two strains. (**C**) Serial dilution spot assay on YPD and YPD with 50 mM hydroxyurea medium for the WT, *dpb3Δ*, *mcm2-3A*, and *dpb3Δ mcm2-3A* strains.

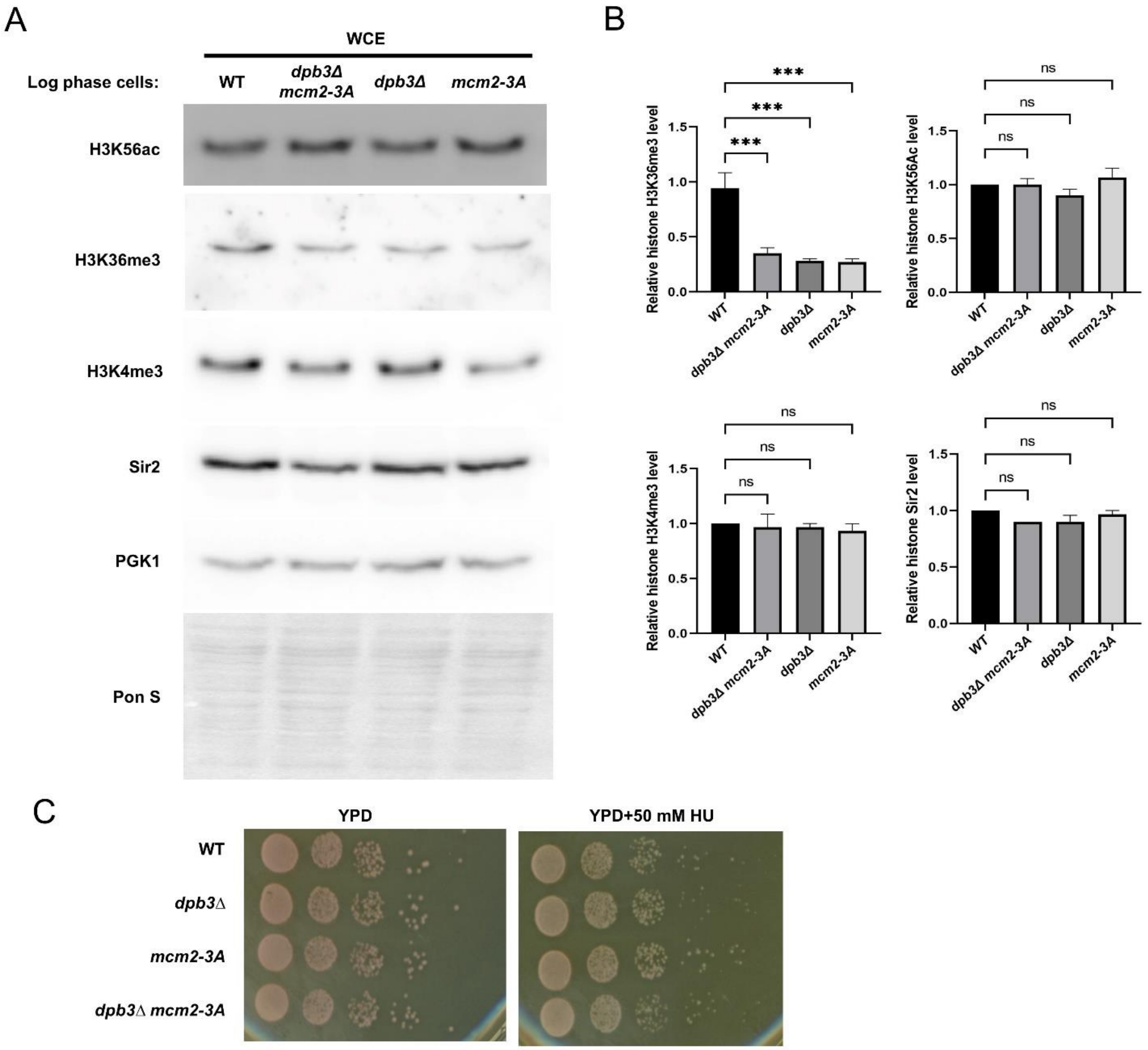

Supplemental Fig 8. Genetic interaction of parental histone chaperone mutants (*dpb3Δ, mcm2-3A*) and new histone chaperone mutants (*cac1Δ, asf1Δ and rtt106Δ*) on Rad52 foci formation.

(**A**) Rad52 foci frequency in WT, the histone dosage regulation mutant (hht2-hhf2Δ), and new histone chaperone mutants (*cac1Δ, asf1Δ* and *rtt106Δ*). The Rad52 foci frequency of *dpb3Δ, mcm2-3A*, and *dpb3Δ mcm2-3A* in *hht2-hhf2Δ* (**B**), *asf1Δ* (**C**), *cac1Δ* (**D**), and *rtt106Δ* (**E**) background. A one-way ANOVA analysis was used for comparing between two strains. Error bars depict standard error of the mean. ns p>0.05, *p < 0.05, **p < 0.01, ***p<0.001, ****p<0.0001.

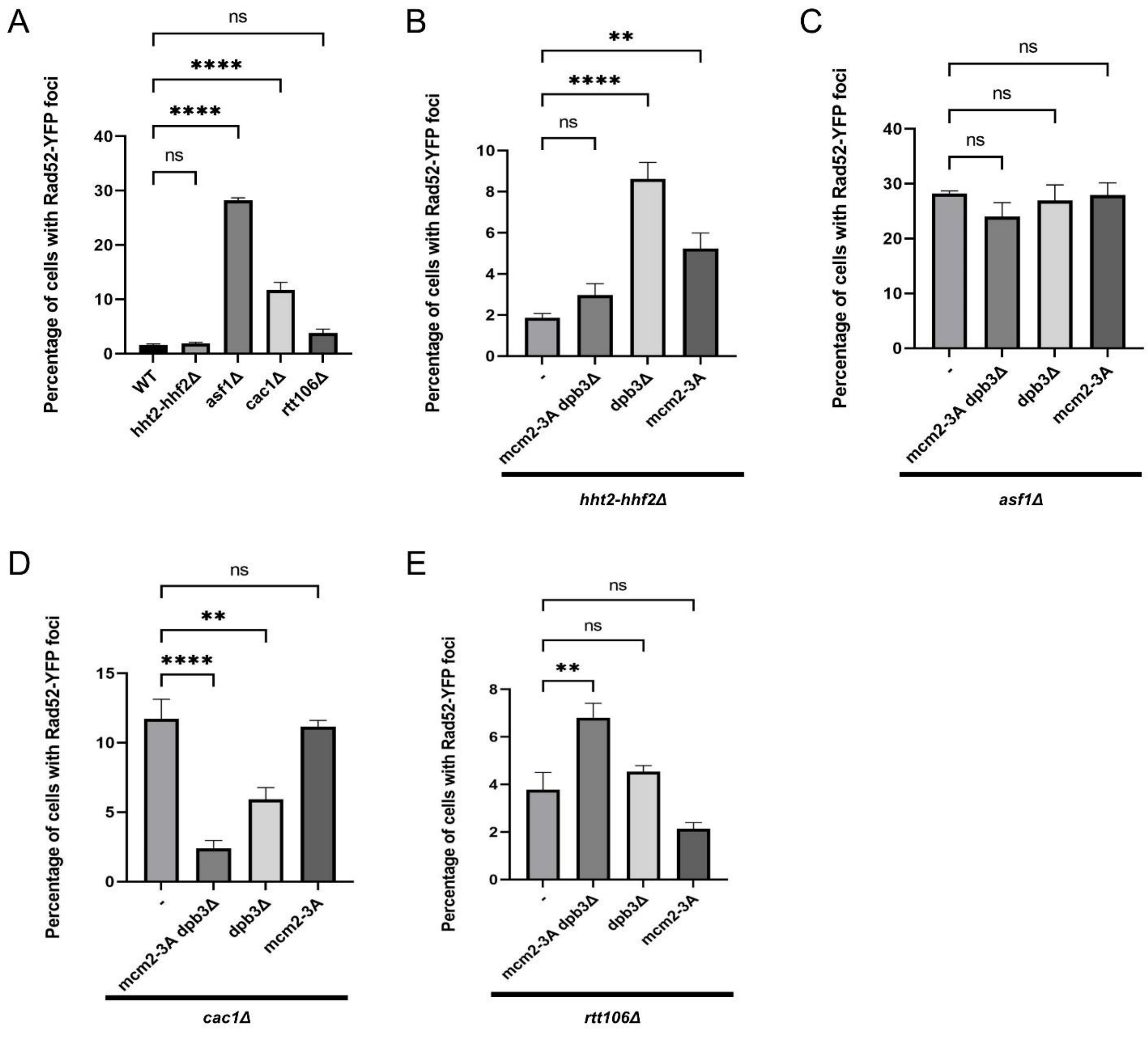

Supplemental Fig 9. Western blot test of rad53-phosphralation and H2AX levels.

(**A**) Western blot test Rad53-phosphralation without/with 0.1% MMS treatment. Rad53 antibody (ab104232, Abcam) was used for Rad53 detection. (**B**) Immunoblot analysis of H2AX levels (anti-γ-H2A antibody (ab15083, Abcam)), a marker of the DNA damage response in yeast, before and after treatment with methylmethane sulphonate (MMS; a DNA damaging agent) in WT, *dpb3Δ*, *mcm2-3A*, and *dpb3Δ mcm2-3A* strains.

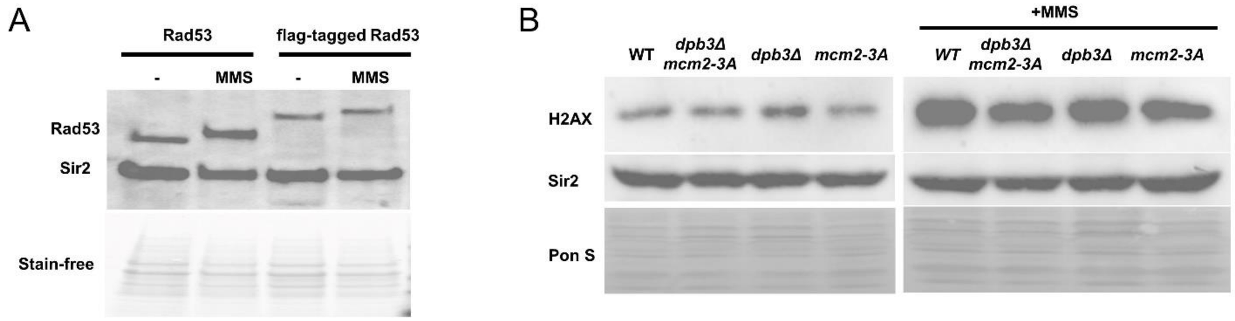

Supplemental Figure 10. Parental histone chaperone mutations increase free histone levels. (**A-B**) Immunoblot of H3, H3K4me3(a marker for chromatin proteins) and PGK1 (a marker for soluble proteins) in the whole cell extract, soluble fraction, and chromatin fraction of WT, *dpb3Δ*, *mcm2-3A*, *dpb3Δ mcm2-3A* and *rad53 Δ* strains. The experiment procedure following Fig 5A except two time points (60minutes and 80minutes). H3K4me3 (ab8580 Abcam); PGK1(ab113687 Abcam) and H3-HA (12CA5 Sigma) were used for Western blot. (**C-D**) Quantitation of soluble H3 and H3K4me3. The signals obtained for soluble histones were normalized to the signals obtained for soluble PGK1 on western blots (C-D). (**E-F**) Soluble H3 and H3K4me3 levels are higher in *dpb3Δ*, *mcm2-3A*, *dpb3Δ mcm2-3A* and *rad53 Δ*. The data shown in comes from average of three independent time points (50 minutes, 60 minutes and 80 minutes) data. Error bars depict standard error of the mean. The signals obtained for soluble histones were normalized to the signals obtained for soluble PGK1 on western blots. Error bars depict standard error of the mean. A one-way ANOVA analysis was used for comparing between two strains. *p < 0.05, **p < 0.01, ***p < 0.001.

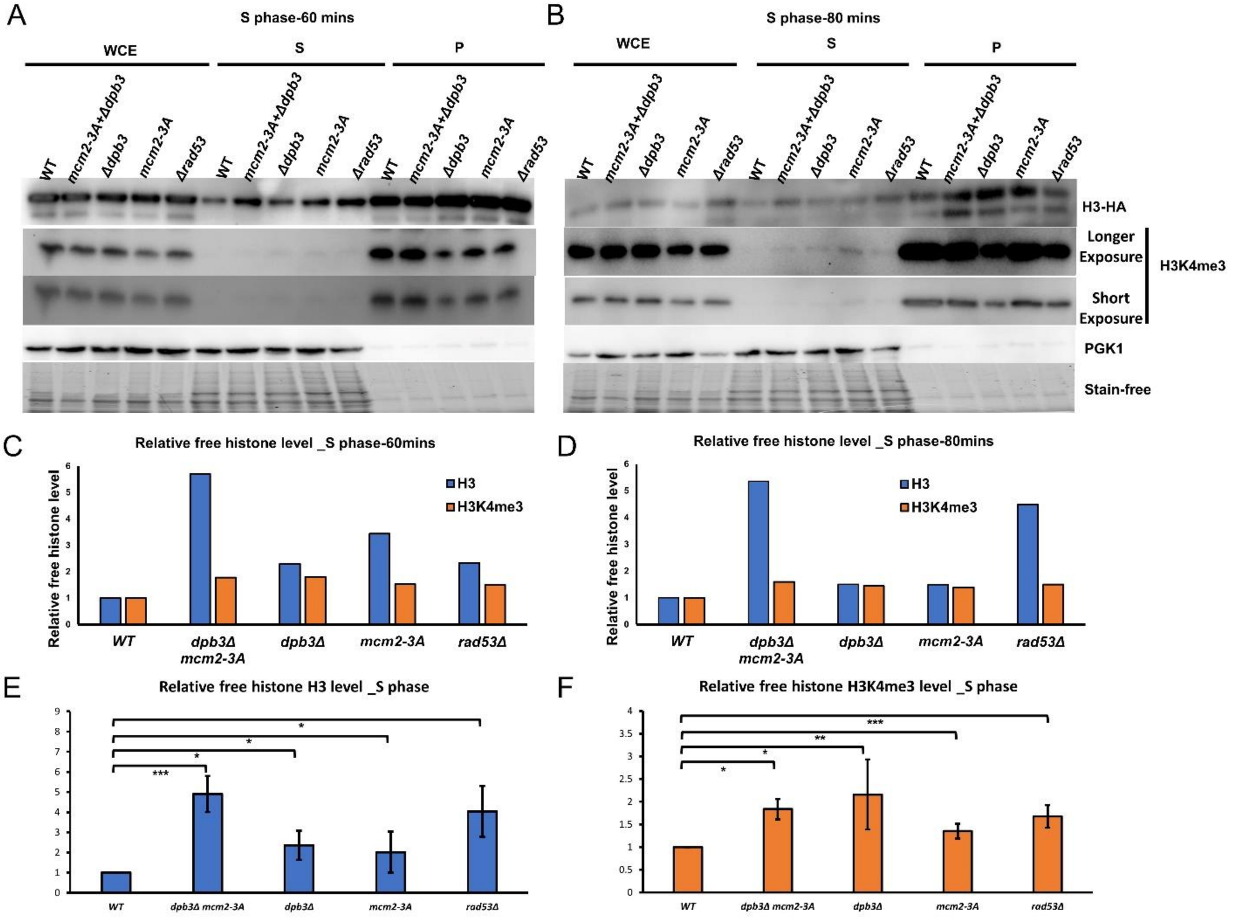

Supplemental Figure 11 Overexpression and deletion of new histone chaperones (*Caf1, Asf1, Rtt106*) on the soluble histone H3 level and HR frequency of *dpb3Δ mcm2-3A* double mutant.

(**A**) Immunoblot of H3 and PGK1 in the whole cell extract, soluble fraction, and chromatin fraction of *dpb3Δ mcm2-3A* strain with overexpressing Caf1, Rtt106 and Asf1. The experimental following **Fig 5A**. The empty-vector control pRS425 (High copy vector) and a plasmid containing all three genes of the Caf-1 complex (pCAF-1, alias pJR3418), ASF1 (pASF1, alias pJR3425), or RTT106 (pRTT106, alias pJR3419) (4) were transformed into *dpb3Δ mcm2-3A* mutant cells. (**B**) Quantitation of soluble H3. The data shown comes from two independent experiments. The signals obtained for soluble histones were normalized to the signals obtained for soluble PGK1 on western blots. Error bars depict standard error of the mean. A one-way ANOVA analysis was used for comparing between two strains. ns: p>0.05. (**C**) Overexpression of the new histone chaperone complex's effect on the HR efficiency of WT. The empty-vector control pRS425 (High copy vector) and a plasmid containing all three genes of the CAF-1 complex (pCAF-1, alias pJR3418), ASF1 (pASF1, alias pJR3425), or RTT106 (pRTT106, alias pJR3419) (42) were transformed into WT. (**D**) Overexpression of the new histone chaperone complexes cannot rescue the HR efficiency of *dpb3Δ mcm2-3A*. The same plasmids used in (C) were used in this panel. (**E**) Deletion of the new histone chaperones on the HR efficiency of WT and *dpb3Δ mcm2-3A* strains. A one-way ANOVA analysis was used for comparing between empty vector and each overexpressed histone chaperone (C and D). ns: p>0.05, **p < 0.01, ****p<0.0001.

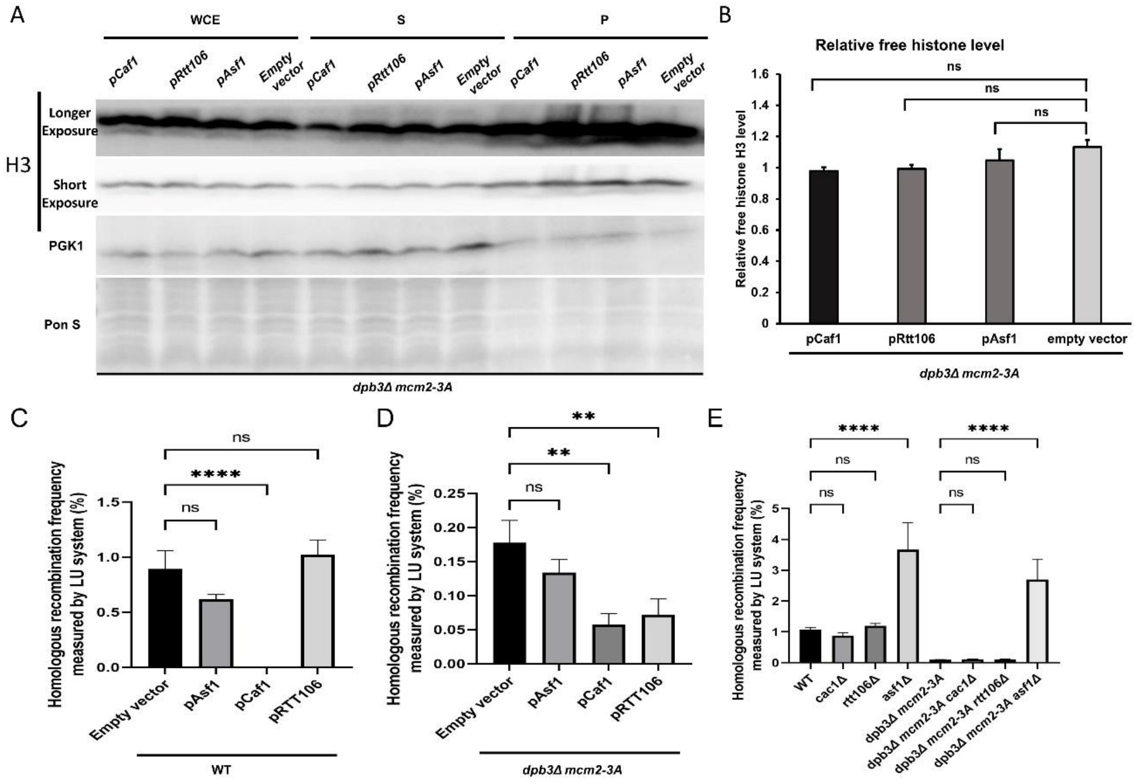

Supplemental Figure 12 Genetic interaction between FACT mutant(*spt16-11*) and *dpb3Δ*, *mcm2-3A*, and *dpb3Δ mcm2-3A*.

(**A-B**) Relative yeast growth quantification from spotting assay. Yeast growth spots were quantified using Image J software using 3rd dilution based on Fig 6F. (**C**) Cell growth and resistance to drugs of *spt16-11* mutation with *dpb3Δ, mcm2-3A*, and *dpb3Δ mcm2-3A*. (**D**) *spt16-11* shows reduced homologous recombination frequency, but does not show any synergetic effect with *dpb3Δ*, *mcm2-3A*, and *dpb3Δ mcm2-3A.*

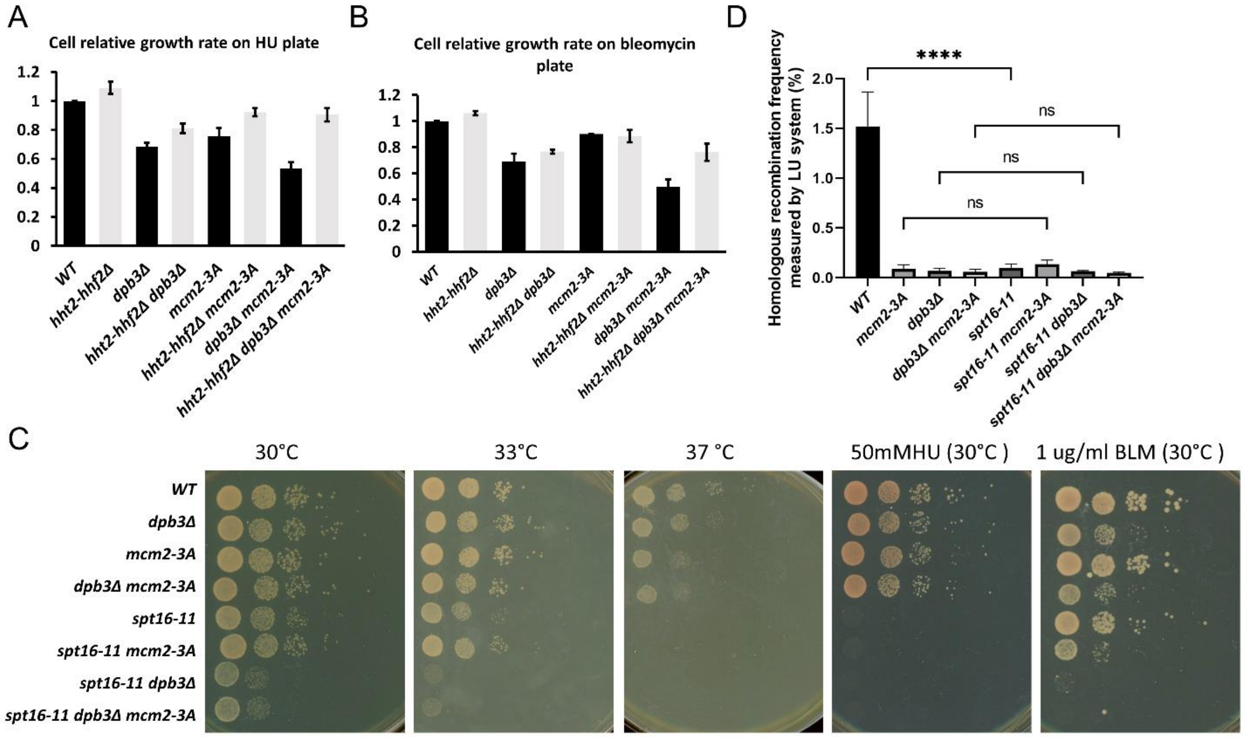

Table S1. Yeast strains used in this study

Strain	Genotype	References
cyc560	*MATA ade2-1 ura3-1 his3-11,15 trp1-1 leu2-3,112 can1-100* + *URA3::BrdU–Inc*	(1,5)
cyc552	*MATA ade2-1 ura3-1 his3-11,15 trp1-1 leu2-3,112 can1-100 mcm2-3A::HphNT* + *URA3::BrdU–Inc*	(5)
cyc602	*MATA ade2-1 ura3-1 his3-11,15 trp1-1 leu2-3,112 can1-100 dpb3Δ::KanMX mcm2-3A::HphNT* + *URA3::BrdU–Inc*	This study
cyc604	*MATA ade2-1 ura3-1 his3-11,15 trp1-1 leu2-3,112 can1-100 dpb3Δ::KanMX* + *URA3::BrdU–Inc*	(1)
cyc575	*MATA ade2-1 ura3-1 his3-11,15 trp1-1 leu2-3,112 can1-100 asf1Δ::KanMX* + *URA3::BrdU–Inc*	This study
cyc579	*MATA ade2-1 ura3-1 his3-11,15 trp1-1 leu2-3,112 can1-100 cac1Δ::LEU2* + *URA3::BrdU–Inc*	This study
cyc712	*MATA ade2-1 ura3-1 his3-11,15 trp1-1 leu2-3,112 can1-100 cac1Δ::LEU2 dpb3Δ::KanMX* + *URA3::BrdU–Inc*	This study
cyc714	*MATA ade2-1 ura3-1 his3-11,15 trp1-1 leu2-3,112 can1-100 cac1Δ::LEU2 mcm2-3A::HphNT* + *URA3::BrdU–Inc*	This study
cyc716	*MATA ade2-1 ura3-1 his3-11,15 trp1-1 leu2-3,112 can1-100 cac1Δ::LEU2 dpb3Δ::KanMX mcm2-3A::HphNT* + *URA3::BrdU–Inc*	This study
cyc718	*MATA ade2-1 ura3-1 his3-11,15 trp1-1 leu2-3,112 can1-100 rtt106Δ::NatMX dpb3Δ::KanMX* + *URA3::BrdU–Inc*	This study
cyc720	*MATA ade2-1 ura3-1 his3-11,15 trp1-1 leu2-3,112 can1-100 rtt106Δ::NatMX mcm2-3A::HphNT* + *URA3::BrdU–Inc*	This study
cyc722	*MATA ade2-1 ura3-1 his3-11,15 trp1-1 leu2-3,112 can1-100 rtt106Δ::NatMX dpb3Δ::KanMX mcm2-3A::HphNT* + *URA3::BrdU–Inc*	This study

cyc724	*MATA ade2-1 ura3-1 his3-11,15 trp1-1 leu2-3,112 can1-100 asf1Δ::KanMX mcm2-3A::HphNT + URA3::BrdU–Inc*	This study
cyc726	*MATA ade2-1 ura3-1 his3-11,15 trp1-1 leu2-3,112 can1-100 asf1Δ::KanMX dpb3Δ::KanMX mcm2-3A::HphNT + URA3::BrdU–Inc*	This study
cyc728	*MATA ade2-1 ura3-1 his3-11,15 trp1-1 leu2-3,112 can1-100 asf1Δ::KanMX dpb3Δ::KanMX + URA3::BrdU–Inc*	This study
cyc730	*MATA ade2-1 ura3-1 his3-11,15 trp1-1 leu2-3,112 can1-100 rtt106Δ::NatMX + URA3::BrdU–Inc*	This study
cyc929	*MATA ade2-1 ura3-1 his3-11,15 trp1-1 leu2-3,112 can1-100 dpb3::NatMX, mcm2-3A::HphNT HHT1-3HA::KanMX+ URA3::BrdU–Inc*	This study
cyc931	*MATA ade2-1 ura3-1 his3-11,15 trp1-1 leu2-3,112 can1-100 dpb3::NatMX HHT1-3HA::KanMX + URA3::BrdU–Inc*	This study
cyc933	*MATA ade2-1 ura3-1 his3-11,15 trp1-1 leu2-3,112 can1-100 mcm2-3A::HphNT HHT1-3HA::KanMX + URA3::BrdU–Inc*	This study
cyc614	*MATA ade2-1 ura3-1 his3-11,15 trp1-1 leu2-3,112 can1-100 HHT1-3HA::KanMX + URA3::BrdU–Inc*	This study
cyc943	*MATA ADE2 ura3-1 his3-11,15 trp1-1 leu2-3,112 can1-100 mcm2-3A::HphNT Rad52-YFP+ URA3::BrdU–Inc*	This study
cyc945	*MATA ADE2 ura3-1 his3-11,15 trp1-1 leu2-3,112 can1-100 dpb3Δ::NatMX bar1 Rad52-YFP+ URA3::BrdU–Inc*	This study
cyc947	*MATA ADE2 ura3-1 his3-11,15 trp1-1 leu2-3,112 can1-100 mcm2-3A::HphNT dpb3Δ::NatMX Rad52-YFP+ URA3::BrdU–Inc*	This study
cyc949	*MATA ADE2 ura3-1 his3-11,15 trp1-1 leu2-3,112 can1-100 Rad52-YFP+ URA3::BrdU–Inc*	This study
cyc939	*MATA ade2-1 his3-11,15 trp1-1 leu2-3,112 can1-100 rad53Δ::HIS HHT1-3HA::KanMX sml1Δ::URA*	This study
cyc949	*MATA ADE2 ura3-1 his3-11,15 trp1-1 leu2-3,112 can1-100 Rad52-YFP+ URA3::BrdU–Inc*	This study

cyc1212	*MATA ADE2 ura3-1 his3-11,15 trp1-1 leu2-3,112 can1-100 mcm2-3A::HphNT asf1Δ::KanMX dpb3Δ::NatMX Rad52-YFP+ URA3::BrdU–Inc*	This study
cyc1214	*MATA ADE2 ura3-1 his3-11,15 trp1-1 leu2-3,112 can1-100 asf1Δ::KanMX dpb3Δ::NatMX Rad52-YFP+ URA3::BrdU–Inc*	This study
cyc1215	*MATB ADE2 ura3-1 his3-11,15 trp1-1 leu2-3,112 can1-100 asf1Δ::KanMX Rad52-YFP+ URA3::BrdU–Inc*	This study
cyc1217	*MATB ADE2 ura3-1 his3-11,15 trp1-1 leu2-3,112 can1-100 hht2-hhf2::TRP1 Rad52-YFP+ URA3::BrdU–Inc*	This study
cyc1218	*MATB ADE2 ura3-1 his3-11,15 trp1-1 leu2-3,112 can1-100 hht2-hhf2::TRP1 mcm2-3A::HphNT dpb3Δ::NatMX Rad52-YFP+ URA3::BrdU–Inc*	This study
cyc1219	*MATB ADE2 ura3-1 his3-11,15 trp1-1 leu2-3,112 can1-100 hht2-hhf2::TRP1 mcm2-3A::HphNT Rad52-YFP+ URA3::BrdU–Inc*	This study
cyc1220	*MATA ADE2 ura3-1 his3-11,15 trp1-1 leu2-3,112 can1-100 hht2-hhf2::TRP1 dpb3Δ::NatMX Rad52-YFP*	This study
cyc1224	*MATB ADE2 ura3-1 his3-11,15 trp1-1 leu2-3,112 can1-100 cac1Δ::LEU2 Rad52-YFP+ URA3::BrdU–Inc*	This study
cyc1227	*MATA ADE2 ura3-1 his3-11,15 trp1-1 leu2-3,112 can1-100 rtt106Δ::KanMX mcm2-3A::HphNT dpb3Δ::NatMX Rad52-YFP+ URA3::BrdU–Inc*	This study
cyc1230	*MATA ADE2 ura3-1 his3-11,15 trp1-1 leu2-3,112 can1-100 rtt106Δ::KanMX Rad52-YFP+ URA3::BrdU–Inc*	This study
cyc1233	*MATA ADE2 ura3-1 his3-11,15 trp1-1 leu2-3,112 can1-100 mcm2-3A::HphNT asf1Δ::KanMX Rad52-YFP+ URA3::BrdU–Inc*	This study
cyc1234	*MATB ADE2 ura3-1 his3-11,15 trp1-1 leu2-3,112 can1-100 rtt106Δ::KanMX dpb3Δ::NatMX Rad52-YFP+ URA3::BrdU–Inc*	This study
cyc1236	*MATA ADE2 ura3-1 his3-11,15 trp1-1 leu2-3,112 can1-100 rtt106Δ::KanMX mcm2-3A::HphNT Rad52-YFP+ URA3::BrdU–Inc*	This study

cyc1237	*MATB ADE2 ura3-1 his3-11,15 trp1-1 leu2-3,112 can1-100 cac1Δ::LEU2 mcm2-3A::HphNT Rad52-YFP+ URA3::BrdU–Inc*	This study
cyc1238	*MATA ADE2 ura3-1 his3-11,15 trp1-1 leu2-3,112 can1-100 cac1Δ::LEU2 dpb3Δ::NatMX Rad52-YFP+ URA3::BrdU–Inc*	This study
cyc1239	*MATA ADE2 ura3-1 his3-11,15 trp1-1 leu2-3,112 can1-100 cac1Δ::LEU2 mcm2-3A::HphNT dpb3Δ::NatMX Rad52-YFP+ URA3::BrdU–Inc*	This study
JRY10790	*MATA lys2 his3-11,15 leu2-3,112 can1-100 hml2alpha::cre ura3Δ::GPDpro-loxP-yEmRFP-CYC1term-hphMX-loxP-yEGFP-ADH1term*	(4)
cyc756	*JRY10790 dpb3Δ::NatMX*	This study
cyc853	*JRY10790 mcm2-3A*	This study
cyc777	*JRY10790 dpb3Δ::NatMX mcm2-3A*	This study
cyc 754	*JRY10790 cac1*Δ*::NatMX*	This study
YLD87	*W303A his3::pRS314-LU-HIS3*	(6)
SK36	*W303A dpb3Δ::KanMX, his3::pRS314-LU-HIS3*	This study
SK37	*W303A mcm2-3A::HphNT, his3::pRS314-LU-HIS3*	This study
SK38	*W303A mcm2-3A -HYg, dpb3Δ::KanMX his3::pRS314-LU-HIS3*	This study
SK149	*W303A dpb3::NatMX, mcm2-3A::HphNT HHT1-3HA::KanMX+ pRS425-Caf1 URA3::BrdU–Inc*	This study
Sk150	*W303A dpb3::NatMX, mcm2-3A::HphNT HHT1-3HA::KanMX+ pRS425-Rtt106 URA3::BrdU–Inc*	This study
SK151	*W303A dpb3::NatMX, mcm2-3A::HphNT HHT1-3HA::KanMX+ pRS425-Asf1 URA3::BrdU–Inc*	This study
SK152	*W303A dpb3::NatMX, mcm2-3A::HphNT HHT1-3HA::KanMX+ pRS425 URA3::BrdU–Inc*	This study
SK165	*W303A dpb3Δ::KanMX mcm2-3A::HphNT his3::pRS314-LU-HIS3+pRS424*	This study

SK166	*W303A dpb3Δ::KanMX mcm2-3A::HphNT his3::pRS314-LU-HIS3+pRS424-Asf1*	This study
Sk167	*W303A dpb3Δ::KanMX mcm2-3A::HphNT his3::pRS314-LU-HIS3+pRS424-Caf1*	This study
SK168	*W303A dpb3Δ::KanMX mcm2-3A::HphNT his3::pRS314-LU-HIS3+pRS424-Rtt106*	This study
SK169	*W303A his3::pRS314-LU-HIS3+pRS424*	This study
SK170	*W303A his3::pRS314-LU-HIS3+pRS424-Asf1*	This study
SK171	*W303A his3::pRS314-LU-HIS3+pRS424-Cac1*	This study
SK172	*W303A his3::pRS314-LU-HIS3+pRS424-Rtt106*	This study
cyc876	*MATA ade2-1 his3-11 trp1-1 leu2-3,112 can1-100, URA3*	This study
cyc881	*MATA ade2-1 his3-11 trp1-1 leu2-3,112 can1-100 dpb3Δ::NatMX URA3*	This study
cyc892	*MATA ade2-1 his3-11 trp1-1 leu2-3,112 can1-100 mcm2-3A URA3*	This study
SK118	*MATA his3::pRS314-LU-HIS3, cac1 Δ::NATMX*	This study
SK120	*MATA his3::pRS314-LU-HIS3, asf1 Δ::NATMX*	This study
SK130	*MATA his3::pRS314-LU-HIS3, Rtt106 Δ::NATMX*	This study
SK122	*MATA mcm2-3A -HYg, dpb3 Δ::KanMX, cac1 Δ::NATMX his3::pRS314-LU-HIS3*	This study
SK131	*MATA mcm2-3A -HYg, dpb3 Δ::KanMX, Rtt106 Δ::NATMX his3::pRS314-LU-HIS3*	This study
SK124	*MATB mcm2-3A -HYg, dpb3 Δ::KanMX, asf1 Δ::NATMX his3::pRS314-LU-HIS3*	This study
Cyc990	*MATA hht2-hhf2::TRP1 his3::pRS314-LU-HIS3*	This study

Cyc1283	*MATB ade2-1 ura3-1 his3-11,15 trp1-1 leu2-3,112 can1-100 dpb3 Δ::KanMX hht2-hhf2::TRP1 his3::pRS314-LU-HIS3*	This study
Cyc1285	*MATB ade2-1 ura3-1 his3-11,15 trp1-1 leu2-3,112 can1-100 mcm2-3A::HPHNT hht2-hhf2::TRP1 his3::pRS314-LU-HIS3*	This study
Cyc1281	*MATB ade2-1 ura3-1 his3-11,15 trp1-1 leu2-3,112 can1-100 dpb3 Δ::KanMX mcm2-3A::HPHNT hht2-hhf2::TRP1 his3::pRS314-LU-HIS3*	This study
cyc1353	*MATA ade2-1 ura3-1 his3-11,15 trp1-1 leu2-3,112 can1-100 spt16-11 his3::pRS314-LU-HIS3*	This study
cyc1355	*MATA ade2-1 ura3-1 his3-11,15 trp1-1 leu2-3,112 can1-100 spt16-11 mcm2-3A::HphNT his3::pRS314-LU-HIS3*	This study
cyc1357	*MATA ade2-1 ura3-1 his3-11,15 trp1-1 leu2-3,112 can1-100 spt16-11 dpb3Δ::KanMX his3::pRS314-LU-HIS3*	This study
cyc1359	*MATA ade2-1 ura3-1 his3-11,15 trp1-1 leu2-3,112 can1-100 spt16-11 dpb3Δ::KanMX mcm2-3A::HphNT his3::pRS314-LU-HIS3*	This study

References

1. Yu, C., Gan, H., Serra-Cardona, A., Zhang, L., Gan, S., Sharma, S., Johansson, E., Chabes, A., Xu, R.M. and Zhang, Z. (2018) A mechanism for preventing asymmetric histone segregation onto replicating DNA strands. *Science (New York, N.Y.)*, **361**, 1386-1389.
2. Han, J., Zhou, H., Horazdovsky, B., Zhang, K., Xu, R.M. and Zhang, Z. (2007) Rtt109 acetylates histone H3 lysine 56 and functions in DNA replication. *Science (New York, N.Y.)*, **315**, 653-655.
3. Zhang, Z., Hayashi, M.K., Merkel, O., Stillman, B. and Xu, R.M. (2002) Structure and function of the BAH-containing domain of Orc1p in epigenetic silencing. *EMBO J*, **21**, 4600-4611.
4. Janke, R., King, G.A., Kupiec, M. and Rine, J. (2018) Pivotal roles of PCNA loading and unloading in heterochromatin function. *Proc Natl Acad Sci U S A*, **115**, E2030-E2039.
5. Gan, H., Serra-Cardona, A., Hua, X., Zhou, H., Labib, K., Yu, C. and Zhang, Z. (2018) The Mcm2-Ctf4-Polalpha Axis Facilitates Parental Histone H3-H4 Transfer to Lagging Strands. *Mol Cell*, **72**, 140-151 e143.
6. Liang, D., Burkhart, S.L., Singh, R.K., Kabbaj, M.H. and Gunjan, A. (2012) Histone dosage regulates DNA damage sensitivity in a checkpoint-independent manner by the homologous recombination pathway. *Nucleic Acids Res*, **40**, 9604-9620.
